# Supplementary material for: FunSpace: A functional and spatial analytic approach to cell imaging data using entropy measures
Source: PLoS Comput Biol. 2023 Sep 27;19(9):e1011490. doi: 10.1371/journal.pcbi.1011490 (PMC10561868; doi:10.1371/journal.pcbi.1011490)
Supplement: S1 Text — (PDF) [file pcbi.1011490.s001.pdf]

Supplementary Information for "FunSpace: A functional and spatial analytic approach to cell imaging data using entropy measures"

Thao Vu[1*], Souvik Seal[1], Tusharkanti Ghosh[1], Mansooreh Ahmadian[1], Julia Wrobel[1,#a], Debashis Ghosh[1*]

[1]Department of Biostatistics and Informatics, University of Colorado Anschutz Medical Campus, Aurora, Colorado, United States of America

[#a]Current Address: Department of Biostatistics and Bioinformatics, Rollins School of Public Health, Emory University, Atlanta, Georgia, United States of America

[*]thao.3.vu@cuanschutz.edu

S.1 Additional details on the implementation of FPCA

We directly implemented the `R` package `fdapace` by Yao et al. [11] to estimate the mean function $\hat{\mu}(s)$ and the covariance surface $\hat{G}(.)$ based on pooled data from all subjects. It is important to note that in the context of imaging data, the images should be collected at the same resolution so that ranges of distance between cells are similar. Furthermore, the images should also share a common set of cell types to ensure a comparable scale of entropy. Briefly, the authors defined the local linear scatterplot smoother for $\mu(s)$ by minimizing

$$\sum_{i=1}^{N}\sum_{k=1}^{K_i}\kappa_1\left(\frac{S_{ik}-s}{h_\mu}\right)\{SPI_{ik}-\beta_0-\beta_1(s-S_{ik})\}^2$$

with respect to β_0 and β_1. And $\kappa_1(.)$ is a kernel function. The estimate of $\mu(s)$ is $\hat{\mu}(s)=\hat{\beta}_0(s)$. The local linear surface smoother for $G(s,t)$ was defined by minimizing

$$\sum_{i=1}^{N}\sum_{1\le k\ne j\le K_i}\kappa_2\left(\frac{S_{ik}-s}{h_G},\frac{S_{ij}-t}{h_G}\right)\times\{G_i(S_{ik},S_{ij})-f(\boldsymbol{\beta},(s,t),(S_{ik},S_{ij}))\}^2$$

where $\kappa_2(.)$ is a bivariate kernel function, $f(\boldsymbol{\beta},(s,t),(S_{ik},S_{ij}))=\beta_0+\beta_{11}(s-S_{ik})+\beta_{12}(t-S_{ij})$. Minimizing the above equation with regard to $\boldsymbol{\beta}=(\beta_0,\beta_{11},\beta_{12})$ yields the estimate $\hat{G}(s,t)=\hat{\beta}_0(s,t)$.

More details on proofs and auxiliary results can be found in [11].

S.2 NSCLC dataset

S.2.1 Additional FPCA results

For each of the five leading FPCs, we overlay the estimated mean function with +/- FPC score multiplying 2 standard deviations (SDs) of the associated score distribution.

Fig S1. First five functional principal components (FPC) obtained from the NSCLC dataset. For each FPC, the mean function is overlaid with +/- FPC score multiplying 2 standard deviations of the associated score distribution.

Fig S2. Histograms of first four FPC scores obtained from the NSCLC dataset. FPC scores were obtained by applying FPCA on the spatial entropy curves from the NSCLC dataset. The scores were centered around 0.

S.2.2 Model outputs on NSCLC dataset

We compared the proposed approach with other diversity measures including Shannon and Simpson indices [7], ecosystem diversity index (EDI) [5], and Mantel correlation [10] with regard to the significant association between the spatial heterogeneity in the TME and overall survival. Specifically, Shannon and Simpson entropy can be computed based on the frequency distribution of CD14+ cells, CD19+ B cells, CD4+ and CD8+ T cells, and CK+ cancer cells [7], without considering for their relative spatial distributions. Additionally, the EDI score can be obtained by first dividing each image into small regions. Based on the cell distribution in each region, Shannon entropy can be obtained. The sequence of Shannon entropy for all regions was fit into a Gaussian mixture model to determine the number of clusters, which was referred to as the EDI. Finally, following Wu et al. [10], a Mantel correlation between the marker expression levels and spatial locations of all cells was calculated for each image. Through a permutation test, the significance level of the Mantel correlation was obtained. The subjects were stratified into geographic diversity (GD) patterns: clustered (p-value < 0.01) vs. random (p-value > 0.01). Below are the outputs for the five models using overall survival as the response.

Spatial entropy

```
Analysis of Deviance Table
```

```
 Cox model: response is  Surv(stime, event)
 Model 1: ~ Age + stage_group + spa_e.1 + spa_e.2
 Model 2: ~ Age + stage_group
   loglik  Chisq Df P(>|Chi|)
1 -383.21
2 -386.80 7.1746  2   0.02767 *
---
Signif. codes:  0 ‘***’ 0.001 ‘**’ 0.01 ‘*’ 0.05 ‘.’ 0.1 ‘ ’ 1
```

Shannon diversity

```
Family: Cox PH
Link function: identity

Formula:
stime ~ Age + stage_group + dvs_shannon

Parametric coefficients:
             Estimate Std. Error z value Pr(>|z|)
Age           0.03756    0.01076   3.490 0.000483 ***
stage_group2  0.10730    0.27913   0.384 0.700686
stage_group3  0.58945    0.28813   2.046 0.040775 *
stage_group4  0.97933    0.61023   1.605 0.108527
dvs_shannon  -0.14540    0.30447  -0.478 0.632967
---
Signif. codes:  0 ‘***’ 0.001 ‘**’ 0.01 ‘*’ 0.05 ‘.’ 0.1 ‘ ’ 1


Deviance explained = 5.56%
-REML = 390.91  Scale est. = 1         n = 153
```

Simpson diversity

```
Family: Cox PH
Link function: identity

Formula:
stime ~ Age + stage_group + dvs_simpson

Parametric coefficients:
             Estimate Std. Error z value Pr(>|z|)
Age           0.03763    0.01079   3.489 0.000486 ***
stage_group2  0.10635    0.27912   0.381 0.703182
stage_group3  0.58758    0.28865   2.036 0.041791 *
stage_group4  0.97638    0.61070   1.599 0.109870
dvs_simpson  -0.23961    0.56568  -0.424 0.671872
---
Signif. codes:  0 ‘***’ 0.001 ‘**’ 0.01 ‘*’ 0.05 ‘.’ 0.1 ‘ ’ 1


Deviance explained = 5.56%
-REML = 390.31  Scale est. = 1         n = 153
```

Ecosystem diversity index (EDI)

```
Family: Cox PH
Link function: identity

Formula:
stime ~ Age + stage_group + EDI

Parametric coefficients:
             Estimate Std. Error z value Pr(>|z|)
Age           0.04015    0.01084   3.702 0.000214 ***
```

```
stage_group2  0.10304    0.27926   0.369 0.712149
stage_group3  0.63581    0.28989   2.193 0.028286 *
stage_group4  1.04329    0.61463   1.697 0.089612 .
EDI           0.08803    0.08408   1.047 0.295101
---
Signif. codes:  0 '***' 0.001 '**' 0.01 '*' 0.05 '.' 0.1 ' ' 1


Deviance explained = 5.89%
-REML = 391.76  Scale est. = 1         n = 153
```

Mantel correlation

```
Call:
coxph(formula = surv_obj ~ lung_group, data = data_combine)

  n= 153, number of events= 89

                   coef exp(coef) se(coef)    z Pr(>|z|)
lung_grouprandom 0.4981    1.6456   0.4256 1.17    0.242

                 exp(coef) exp(-coef) lower .95 upper .95
lung_grouprandom     1.646     0.6077    0.7146      3.79

Concordance= 0.512  (se = 0.012 )
Likelihood ratio test= 1.19  on 1 df,   p=0.3
Wald test            = 1.37  on 1 df,   p=0.2
Score (logrank) test = 1.4  on 1 df,   p=0.2
```

Fig S3. Kaplan–Meier curves for the overall survival probability from the NSCLC dataset, stratified using the Mantel correlation.. Subjects were classified as clustered vs. random based on the permutation test of the Mantel correlation. P-value of 0.24 indicates non-significant difference in survival probability in two groups.

S.3 Model outputs on TNBC dataset

Spatial entropy

```
Analysis of Deviance Table
 Cox model: response is  Surv(stime, event)
 Model 1: ~ Age + spa_e.1 + spa_e.2 + spa_e.3
 Model 2: ~ Age
   loglik  Chisq Df P(>|Chi|)
1 -35.941
2 -39.942 8.0029  3   0.04595 *
---
Signif. codes:  0 ‘***’ 0.001 ‘**’ 0.01 ‘*’ 0.05 ‘.’ 0.1 ‘ ’ 1
```

Shannon diversity

```
Family: Cox PH
Link function: identity


Formula:
stime ~ Age + shannon


Parametric coefficients:
        Estimate Std. Error z value Pr(>|z|)
Age     -0.02302    0.02203  -1.045    0.296
shannon -2.46494    2.06601  -1.193    0.233



Deviance explained = 3.74%
-REML = 40.562  Scale est. = 1         n = 33
```

Simpson diversity

```
Family: Cox PH
```

```
Link function: identity


Formula:
stime ~ Age + simpson


Parametric coefficients:
        Estimate Std. Error z value Pr(>|z|)
Age     -0.02339    0.02214  -1.056    0.291
simpson -3.01707    2.42819  -1.243    0.214



Deviance explained = 3.98%
-REML = 40.332  Scale est. = 1         n = 33
```

Ecosystem diversity index (EDI)

```
Family: Cox PH
Link function: identity


Formula:
stime ~ Age + EDI


Parametric coefficients:
    Estimate Std. Error z value Pr(>|z|)
Age -0.01836    0.02023  -0.907    0.364
EDI  0.04109    0.22071   0.186    0.852



Deviance explained = 1.38%
-REML = 43.499  Scale est. = 1         n = 33
```

Mantel correlation

```
Call:
coxph(formula = surv_obj ~ ITH, data = data_combine)

  n= 33, number of events= 13

            coef exp(coef) se(coef)     z Pr(>|z|)
ITHrandom 0.2424    1.2744   0.5605 0.433    0.665

          exp(coef) exp(-coef) lower .95 upper .95
ITHrandom     1.274     0.7847    0.4248     3.823

Concordance= 0.502  (se = 0.073 )
Likelihood ratio test= 0.19  on 1 df,   p=0.7
Wald test            = 0.19  on 1 df,   p=0.7
Score (logrank) test = 0.19  on 1 df,   p=0.7
```

Fig S4. Kaplan–Meier curves for the overall survival probability from the TNBC dataset, stratified using the Mantel correlation.. Subjects were classified as clustered vs. random based on the permutation test of the Mantel correlation. P-value of 0.66 indicates non-significant difference in survival probability in two groups.

S.4 Analysis of ovarian cancer data

S.4.1 Data

Tissue microarray (TMA) slides of 132 ovarian cancer patients were stained with antibodies specific for CD3, CD4, CD8, CD19, CD68, cytokeratin, Ki67, pStat, and IER3. The slides were imaged using Vectra 3.0 microscope (Akoya Biosystems) and then segmented and phenotyped using the `inForm` software. More details can be found in [3]. Within the cohort, we excluded 18 patients from the analysis due to missingness of clinical information.

Fig S5. Ovarian cancer dataset: (A) Representative images with distribution of immune cells including CD19+ B cells, CD4+ T cells, CD8+ T cells, CD68+ macrophages, and CK+. (B) Spatial entropy of the five cell types as a function of inter-cell distances.

Fig S6. FPCA results from SPI curves in ovarian cancer dataset. (A) Mean function. (B) First two eigenfunctions.

S.4.2 Results

Motivated by recent studies on the immune responses in the ovarian TME [1, 6], we explored the spatial heterogeneity in various immune cell subsets such as T cells, B cells, and tumor-associated macrophages, in relataive to CK+ tumor cells. Fig S5A illustrates distribution of four different immune cell types including CD19+ B cells, CD4+ T cells, CD8+ T cells, CD68+ macrophages, and CK+ cells in four representative individuals. Utilizing the entropy measures introduced in Section 2.1.1 for $I = 5$ categories, the heterogeneity in spatial distributions of these cell types was captured for each individual image. Fig S5B shows spatial entropy measures as a function of inter-cell distances, which we call SPI curves, for all 114 subjects . In particular, there was a high level of variation in spatial entropy values at distances less than 200 μm at which some individuals expressed high entropy values while others had values close to zero.

The SPI curves were used as input for the FPCA analysis defined in Section 2.1.2 to obtain the estimated FPC scores. Fig S6 shows the estimated mean function (A) and the first two eigenfunctions (B). The estimated mean function reflected the overall trend starting at relatively high entropy values at short distances ($\leq 200\ \mu m$), then dropping off close to zero as the distances increase. The first eigenfunction showed similar trend to the mean function while the second one expressed a contrast in spatial entropy values between distances of $< 120\ \mu m$ vs. $> 120\ \mu m$. The first two FPC scores accounted for 97.6% of the total variation.

By fitting the two selected FPC scores directly into model (4) in Section 2.1.3, we investigated the relationship between the heterogeneity in spatial distributions of immune cells and CK+ cells within the TME and survival outcome, in addition to subject age. Full and restricted models were fit to test the hypothesis Section 2.1.3. We obtained the p-value of 0.15, suggesting non-significant effect of spatial heterogeneity in TME immune composition on the overall survival.

Spatial entropy

```
Analysis of Deviance Table
```

```
 Cox model: response is  Surv(stime, event)
 Model 1: ~ Age + spi.1 + spi.2
 Model 2: ~ Age
   loglik  Chisq Df Pr(>|Chi|)
1 -280.69
2 -282.56 3.7391  2     0.1542
```

Shannon diversity

```
Family: Cox PH
Link function: identity

Formula:
stime ~ Age + shannon

Parametric coefficients:
        Estimate Std. Error z value Pr(>|z|)
Age      0.01749    0.01158   1.511    0.131
shannon -0.32173    0.36831  -0.874    0.382


Deviance explained = 1.36%
-REML = 286.12  Scale est. = 1         n = 114
```

Simpson diversity

```
Family: Cox PH
Link function: identity

Formula:
stime ~ Age + simpson

Parametric coefficients:
```

```
        Estimate Std. Error z value Pr(>|z|)
Age      0.01728    0.01161   1.488    0.137
simpson -0.49627    0.68410  -0.725    0.468


Deviance explained = 1.28%
-REML = 285.62  Scale est. = 1         n = 114
```

Ecosystem diversity index (EDI)

```
Family: Cox PH
Link function: identity

Formula:
stime ~ Age + EDI

Parametric coefficients:
    Estimate Std. Error z value Pr(>|z|)
Age  0.01482    0.01152   1.287    0.198
EDI  0.05492    0.09391   0.585    0.559


Deviance explained = 1.21%
-REML = 287.71  Scale est. = 1         n = 114
```

In addition to spatial entropy, we also performed similar association tests using other diversity metrics including Shannon index, Simpson index, and ecosystem diversity index (EDI) [5]. Corresponding model outputs are displayed above. None of the metrics identified significant association between spatial heterogeneity in the TME cellular composition and overall survival.

Fig S7. IHC COVID-19 dataset: (A) Representative images with distribution of 17 different cell types (e.g., B cells, CD4 T cells, etc.) (B) Spatial entropy of all cell types as a function of inter-cell distances.

Fig S8. FPCA results from SPI curves in IHC COVID-19 dataset. (A) Mean function. (B) First three eigenfunctions.

S.5 Analysis of COVID-19 progression IHC data

S.5.1 Data

We used an immunohistochemistry (IHC) data collected from a cohort of 23 patients available at https://doi.org/10.5281/zenodo.4633905 to explore the spatial organization of the cellular environment of the lung to gain insights into the progression of lung disease. The cohort included four individuals who died with acute respiratory distress syndrome (ARDS), two with influenza, three with pneumonia, four early-stage COVID-19, six late-stage COVID-19, and four without lung disease. Details of the immunohistochemical staining of lung tissue, cell segmentation, and cell-type identification can be found in [8]. Each acquired image for each individual consisted of multiple region segments (5 to 14) corresponding to different regions of the same tissue sample. Due to the sparsity in terms of number of cells for some regions, we decided to select the regions with the maximum number of cells to represent each subject. Please refer to [8] for more details.

S.5.2 Results

With survival outcome not being available, in this analysis, we focused on using spatial heterogeneity among 17 cell types in the cellular microenvironment of the lung to classify different stages of COVID-19 progression. Fig S7A shows distributions of 17 different cell types (e.g., B cells, CD4 T cells, etc.) in four representative individuals. Utilizing the spatial entropy measures introduced in Section 2.1 for $I = 17$ categories, the heterogeneity in spatial distributions of these cell types was captured for each individual image. Fig S7B shows spatial entropy measures as a function of inter-cell distances, which we refer to as SPI curves, for all 23 subjects . Specifically, higher variability in the entropy values were observed at short distances (≤ 250 pixels) and the variation reduced as distances increased.

The SPI curves were used as input for the FPCA analysis defined in Section 2.2 to obtain the estimated FPC scores. Fig S8 shows the estimated mean function (A) and the first three eigenfunctions (B). The estimated mean function displayed the overall trend starting at relatively high entropy values at short distances (≤ 250 pixels), then dropping off close to zero as distances increased. The first

eigenfunction showed similar trend to the mean function while the second and third eigenfunctions highlighted a contrast in spatial entropy values between distances of ≤ 100 pixels vs. ≥ 100 pixels.

By fitting the three selected FPC scores as covariates of a multinomial regression model [2, 4], we investigated how well the heterogeneity in spatial distributions of different cell types was to classify individuals at different COVID-19 progression stages. The classification model that accounted for the spatial heterogeneity via spatial entropy yielded an accuracy of 0.61. When Shannon and Simpson diversity indexes were used instead in the classification model, the accuracy was 0.39.

Spatial entropy

	Prediction					
Truth	ARDS	COVID19_early	COVID19_late	Flu	Healthy	Pneumonia
ARDS	3	0	1	0	0	0
COVID19_early	0	3	1	0	0	0
COVID19_late	2	0	3	0	1	0
Flu	1	0	0	1	0	0
Healthy	0	0	1	0	2	1
Pneumonia	0	0	0	0	1	2

Shannon diversity

	Prediction					
Truth	ARDS	COVID19_early	COVID19_late	Flu	Healthy	Pneumonia
ARDS	0	0	4	0	0	0
COVID19_early	0	0	1	0	2	1
COVID19_late	0	0	6	0	0	0
Flu	0	0	2	0	0	0
Healthy	0	0	2	0	2	0
Pneumonia	0	0	2	0	0	1

Simpson diversity

	Prediction					
Truth	ARDS	COVID19_early	COVID19_late	Flu	Healthy	Pneumonia

ARDS	0	0	4	0	0	0
COVID19_early	0	0	2	0	1	1
COVID19_late	0	0	6	0	0	0
Flu	0	0	2	0	0	0
Healthy	0	0	2	0	2	0
Pneumonia	0	0	2	0	0	1

S.6 Simulation studies

S.6.1 Design

We generated subject-specific entropy curves by adding noise to the reference curves (Fig. 6C). We considered three scenarios (Fig S9) corresponding to the three levels of additive noise: small, medium, and large.

Fig S9. Simulation scenarios: (A) low additive noise. (B) medium additive noise. (C) large additive noise. Three levels of noise were added to the reference SPI curves (clustered vs. random) to generate subject-specific SPI curves.

S.6.2 Additional Simulation 1

We computed reference Shannon entropy values [9] with respect to the two simulation spatial configurations in Fig 6. To generate subject-specific Shannon entropy for each simulated image, we introduced three levels of additive noise (small, medium, and large) following Gaussian distributions with mean 0 and standard deviations of 0.25, 0.5, and 1, respectively. At each level of noise, we generated datasets of different sizes (N = 100, 200, 500, and 1000). Following the simulation in Section 4, we partitioned each dataset into training (75%) and testing (25%) sets. Four models were fit using the training set:(1) a model accounting for both clinical predictor and spatial heterogeneity (2) a model accounting for only spatial heterogeneity, (3) a model accounting for only clinical predictor, and (4) a model accounting for spatial heterogeneity using Shannon entropy. We evaluated the predictive performance of the four different models on the testing sets using normalized root mean squared error (NRMSE). We repeated the simulation for 100 iterations and recorded the average NRMSE for each of the four models across four sample sizes: N = 100, 200, 500, 1000 in Table A. The variation of NRMSEs decreased as sample sizes increased from N = 100 to N = 1000. Note that when there was a low level of

subject-specific variation (Fig S9A), the separation between the two spatial configurations was clear. As a result, all four models performed almost equivalently, particularly at the largest sample size N = 1000. However, when the additive noise was greatly increased, the difference in the spatial entropy curves across the two patterns was not as pronounced (Fig S9C). In the extreme case at N = 100, by accounting for the impact of the heterogeneity in the spatial configurations, models (1) and (2) yielded significantly smaller NRMSEs compared to the remaining two models. Without accounting for the spatial information, model (4) that used Shannon entropy as a predictor, exhibited relatively higher NRMSEs. However, as the sample size increased, the benefit of accounting for spatial impact became less noticeable. In other words, there was no significant difference in the performance of the four models.

Table A. Additional Simulation 1. Normalized root mean squared errors (NRMSE) across four different models, with four sample sizes (N = 100, 200, 500, 1000), and at three levels of additive noise (small, medium, and large). For each setting, we performed 100 simulations and recorded the corresponding standard deviations in parentheses.

Noise level	Model	N=100	N=200	N=500	N=1000
Small	(1)	0.89 (1.05)	1.04 (0.04)	1.10 (0.02)	1.12 (0.02)
	(2)	1.19 (1.18)	1.10 (0.03)	1.12 (0.02)	1.13 (0.01)
	(3)	1.02 (0.06)	1.06 (0.03)	1.10 (0.02)	1.11 (0.01)
	(4)	1.27 (0.23)	1.15 (0.05)	1.14 (0.03)	1.13 (0.02)
Medium	(1)	0.39 (0.18)	1.04 (0.04)	1.11 (0.02)	1.12 (0.02)
	(2)	0.66 (0.15)	1.10 (0.04)	1.13 (0.02)	1.14 (0.01)
	(3)	1.02 (0.07)	1.07 (0.03)	1.11 (0.02)	1.12 (0.01)
	(4)	1.18 (0.09)	1.14 (0.03)	1.14 (0.02)	1.14 (0.01)
Large	(1)	0.41 (0.14)	1.04 (0.05)	1.12 (0.03)	1.14 (0.02)
	(2)	0.70 (0.10)	1.11 (0.04)	1.15 (0.02)	1.16 (0.02)
	(3)	1.03 (0.07)	1.08 (0.04)	1.13 (0.02)	1.14 (0.02)
	(4)	1.17 (0.06)	1.16 (0.03)	1.16 (0.02)	1.16 (0.02)

S.6.3 Additional Simulation 2

Using a similar simulation setup, we assumed that there were two groups of subjects. The number of subjects per group followed a binomial distribution with a probability of 0.5, i.e., $N_g \sim Binom(N = 100, 0.5)$ with $g = 1, 2$. We considered two spatial configurations: clustered vs. random (Fig. 6 a,b), corresponding to $g = 1, 2$, respectively. However, rather than generating spatial entropy curves specific to each subject by adding noise to the reference curves, we directly simulated subject-specific point patterns. Specifically, we simulated the number of cells for each cell type: CD14+, CD19+, CD4+, CD8+, and CK+, from a negative binomial distribution with the mean and dispersion parameter randomly selected from two ranges [200:500] and [1:3], respectively. Next, based on the previous clustered configuration (Fig. 6A), we varied the x-coordinates of some randomly selected cells to create overlapping between clusters of cell types. Fig S10 shows two representative simulated images.

The rationale behind this setup was to introduce variations in both the total number of cells per cell type and the spatial information. We then computed spatial entropy curve and Shannon entropy for each simulated image. Following the procedure described above, the dataset was partitioned into training (75%) and testing (25%) sets. We fit four models on the training set: (1) a model accounting for both clinical predictor and spatial heterogeneity (2) a model accounting for only spatial heterogeneity, (3) a model accounting for only clinical predictor, and (4) a model accounting for spatial heterogeneity using Shannon entropy. Given the computational intensity, we performed 45 simulations at N = 100 and recorded the NRMSE distribution for each model in Table B. As expected, model (1) that accounted for both clinical effect and spatial heterogeneity, exhibited significantly smaller NRMSEs than the other three models. Notably, model (4), which incorporated Shannon entropy to capture cell type diversity but not their relative spatial distribution, resulted in the largest NRMSEs.

Fig S10. Representative simulated cell distributions for additional simulation 2. The number of cells for each cell type was simulated from a negative binomial distribution with the mean and dispersion parameter randomly selected from two ranges [200:500] and [1:3], respectively. The x-coordinates of some randomly selected cell were varied to create overlapping between clusters of cell types.

Table B. Additional simulation 2. Normalized root mean squared errors (NRMSE) across four different models at N = 100. We performed 45 simulations and recorded the corresponding standard deviations in parentheses.

Model	N=100
(1)	0.50 (0.25)
(2)	0.79 (0.18)
(3)	1.04 (1.09)
(4)	2.19 (1.26)

S.7 Sensitivity Analysis

S.7.1 Rotation

We explored the impact of rotation on the spatial entropy results. Specifically, we rotated the clustered point pattern introduced in Fig 6A at various angles to obverse any potential changes in the resulting spatial entropy. Fig S11 shows the original point pattern and its rotations at 180°, 90°, 60°, 45°, and 36°. At each rotation, pairwise distances between all cells were calculated based on their corresponding x- and y-coordinates. Distance ranges were subsequently computed. Finally, the spatial entropy curve across all distance ranges was obtained. Fig S12 displays the corresponding spatial entropy which remains unchanged across the five rotations.

Fig S11. Configuration Rotation. The simulated clustered point pattern (i.e., original) introduced in Fig 6A was rotated at various angles including 180°, 90°, 60°, 45°, and 36°.

Fig S12. SPI under different configuration rotations. At each rotation, pairwise distances between all cells were calculated based on their corresponding x- and y-coordinates. Distance ranges were subsequently computed. Finally, the spatial entropy curve across all distance ranges was obtained.

S.7.2 Number of distance ranges: K

We explore the impact of varying the total number of distance breaks, denoted by K, on the resulting spatial entropy curves. Particularly, we utilized the simulated clustered configuration depicted in Fig 6A as a case study. We computed the pairwise Euclidean distances between all points in the pattern based on their xy-coordinates. As the general focus was on local cell-to-cell interactions, we specifically considered short pairwise distances. We set the maximum distance range for consideration to be the median of the distance distribution, denoted as d_K. We then generated a sequence of distance breaks of length K by linearly decreasing from d_K to 0 on a log scale. Fig S13 shows the spatial entropy curves corresponding to different K values which were incremented from 25 to 100 in steps of 5. The distance ranges became relatively larger as K decreased. We observed a higher number of co-occurrences between cell types within each range. This resulted in smoother spatial entropy curves. On the contrary, with an increase in K, the spatial entropy curves exhibited greater fluctuations at shorter distances.

Fig S13. SPI under K different distance ranges. K values were incremented from 25 to 100 in steps of 5. At each K, a sequence of distance breaks was generated by linearly decreasing from d_K to 0 on a log scale. Distance ranges were subsequently computed. Finally, the spatial entropy curve across all distance ranges was obtained.

Fig S14. Empirical distribution of $p(z_r|w_k)$. One representative image in the NSCLC dataset was used for demonstration. Based on pairwise distances between cells, we generated $K = 50$ distance breaks w_k for $k = 1, \ldots, K$. At each w_k, the co-occurrences between cell types $p(z_r|w_k)$ were obtained. We then built this panel of histograms using the relative frequencies of the cell type co-occurrences.

S.8 Illustration of spatial entropy computation

In this section, we present a quick illustration for the spatial entropy calculation using the random variables Y, Z, and W that were defined in the main text. To accomplish this, we simulated a point pattern (Fig S15) consisting of 30 cells, with 10 cells per each type A, B, and C, which were represented by Y random variable in the text. Shannon entropy can be calculated as

Fig S15. Illustration for SPI calculation. A point pattern consisting of 30 cells, with 10 cells per each type A, B, and C, was simulated. Circles of radius 0.25 were drawn around each point to identify co-occurrences of cell types with the first distance range $w_1 = (0, 0.25]$. Specifically, there were 1 AA, 1 BB, 3 CC, 0 AB, 0 AC, and 0 BC.

$$H(Y) = 3 \times \frac{1}{3} \log(3) = 1.1$$

We defined co-occurrences (denoted by Z random variable) as all possible combinations of the cell types of any two cells, including AA, BB, CC, AB, AC, and BC. Based on the pairwise distances between all cells in the pattern, we generated distance breaks d_k for $k = 1, \ldots, K$ of length $K = 3$. Consequently, we had three contiguous distance ranges (i.e., W random variable) of $w_1 = (0, 0.25]$, $w_2 = (0.25, 1.12]$, and $w_3 = (1.12, 7]$.

For simplicity, we used the first range, $w_1 = (0, 0.25]$, for illustrative purposes. We drew circles of radius 0.25 around each point to identify any co-occurrences of cell types that took place within the distance range $w_1 = (0, 0.25]$. From Fig S15, we can see that there are 1 AA, 1 BB, 3 CC, 0 AB, 0 AC, and 0 BC. As a result, the corresponding relative frequencies were $p(z_{AA}|w_1) = 0.2$, $p(z_{BB}|w_1) = 0.2$, $p(z_{CC}|w_1) = 0.6$, and $p(z_{AB}|w_1) = p(z_{AC}|w_1) = p(z_{BC}|w_1) = 0$. Additionally, when space was not considered, there were 45 AA, 45 BB, 45 CC, 100 AB, 100 AC, and 100 CC. In other words, the overall distribution of co-occurrences,i.e., $p(Z)$ was $p(z_{AA}) = p(z_{BB}) = p(z_{CC}) = 0.103$, $p(z_{AB}) = p(z_{AC}) = p(z_{BC}) = 0.23$. Thus, the spatial entropy at $w_1 = (0, 0.25]$ can be calculated as

$$\begin{aligned} SPI_1 = SPI(Z|w_1) &= 0.2 \log\left(\frac{0.2}{0.103}\right) + 0.2 \log\left(\frac{0.2}{0.103}\right) + 0.6 \log\left(\frac{0.6}{0.103}\right) + 0 + 0 + 0 \\ &= 1.3 \end{aligned}$$

Similar procedure can be performed to compute SPI at w_2 and w_3. Here, SPI_k for $k = 1, 2, 3$ represents entropy due to space.

Residual entropy can be calculated as

$$\begin{aligned} H_1^W = H^W(Z|w_1) &= 0.2 \log\left(\frac{1}{0.2}\right) + 0.2 \log\left(\frac{1}{0.2}\right) + 0.6 \log\left(\frac{1}{0.6}\right) + 0 + 0 + 0 \\ &= 0.95 \end{aligned}$$

References

1. Denisa Baci, Annalisa Bosi, Matteo Gallazzi, Manuela Rizzi, Douglas M Noonan, Alessandro Poggi, Antonino Bruno, and Lorenzo Mortara. The ovarian cancer tumor immune microenvironment (time) as target for therapy: a focus on innate immunity cells as therapeutic effectors. *International Journal of Molecular Sciences*, 21(9):3125, 2020.

2. David W Hosmer Jr, Stanley Lemeshow, and Rodney X Sturdivant. *Applied logistic regression*, volume 398. John Wiley & Sons, 2013.

3. Kimberly R Jordan, Matthew J Sikora, Jill E Slansky, Angela Minic, Jennifer K Richer, Marisa R Moroney, Junxiao Hu, Rebecca J Wolsky, Zachary L Watson, Tomomi M Yamamoto, et al. The capacity of the ovarian cancer tumor microenvironment to integrate inflammation signaling conveys a shorter disease-free interval. *Clinical Cancer Research*, 26(23):6362–6373, 2020.

4. Scott Menard. Coefficients of determination for multiple logistic regression analysis. *The American Statistician*, 54(1):17–24, 2000.

5. Rachael Natrajan, Heba Sailem, Faraz K Mardakheh, Mar Arias Garcia, Christopher J Tape, Mitch Dowsett, Chris Bakal, and Yinyin Yuan. Microenvironmental heterogeneity parallels breast cancer progression: a histology–genomic integration analysis. *PLoS medicine*, 13(2):e1001961, 2016.

6. Franklin Ning, Christopher B Cole, and Christina M Annunziata. Driving immune responses in the ovarian tumor microenvironment. *Frontiers in Oncology*, page 3060, 2021.

7. So Yeon Park, Mithat Gönen, Hee Jung Kim, Franziska Michor, Kornelia Polyak, et al. Cellular and genetic diversity in the progression of in situ human breast carcinomas to an invasive phenotype. *The Journal of clinical investigation*, 120(2):636–644, 2010.

8. André F Rendeiro, Hiranmayi Ravichandran, Yaron Bram, Vasuretha Chandar, Junbum Kim, Cem Meydan, Jiwoon Park, Jonathan Foox, Tyler Hether, Sarah Warren, et al. The spatial landscape of lung pathology during covid-19 progression. *Nature*, 593(7860):564–569, 2021.

9. Claude Elwood Shannon. A mathematical theory of communication. *The Bell system technical journal*, 27(3):379–423, 1948.

10. Hua-Jun Wu, Daniel Temko, Zoltan Maliga, Andre L Moreira, Emi Sei, Darlan Conterno Minussi, Jamie Dean, Charlotte Lee, Qiong Xu, Guillaume Hochart, et al. Spatial intra-tumor heterogeneity is associated with survival of lung adenocarcinoma patients. *Cell Genomics*, 2(8):100165, 2022.

11. Fang Yao, Hans-Georg Müller, and Jane-Ling Wang. Functional data analysis for sparse longitudinal data. *Journal of the American statistical association*, 100(470):577–590, 2005.
