# Supplementary figures and images for: FunSpace: A functional and spatial analytic approach to cell imaging data using entropy measures
Source: PLoS Comput Biol. 2023 Sep 27;19(9):e1011490. doi: 10.1371/journal.pcbi.1011490 (PMC10561868; doi:10.1371/journal.pcbi.1011490)

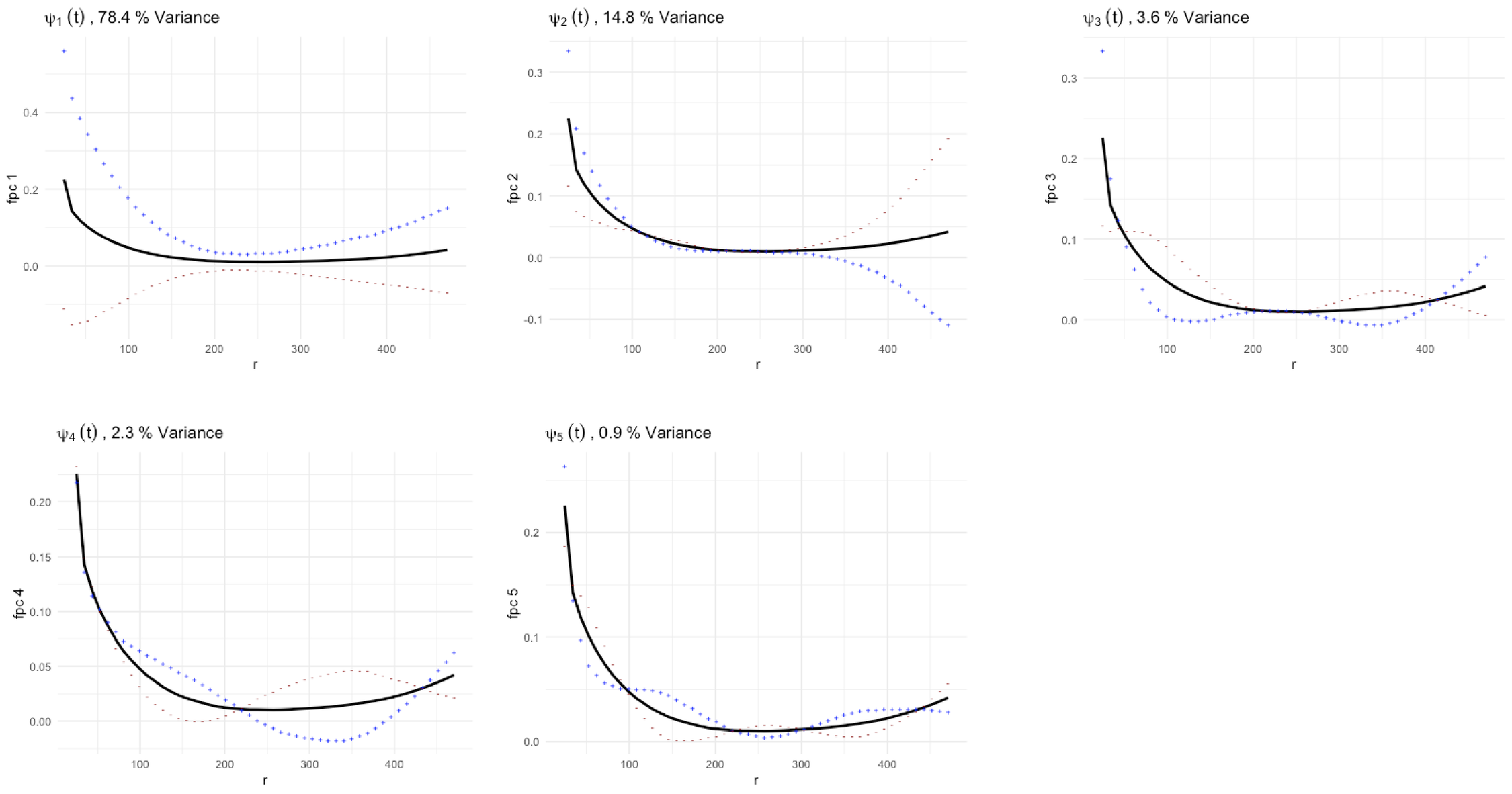

Supplement: S1 Fig — For each FPC, the mean function is overlaid with +/- FPC score multiplying 2 standard deviations of the associated score distribution. (TIF) [file pcbi.1011490.s002.tif]

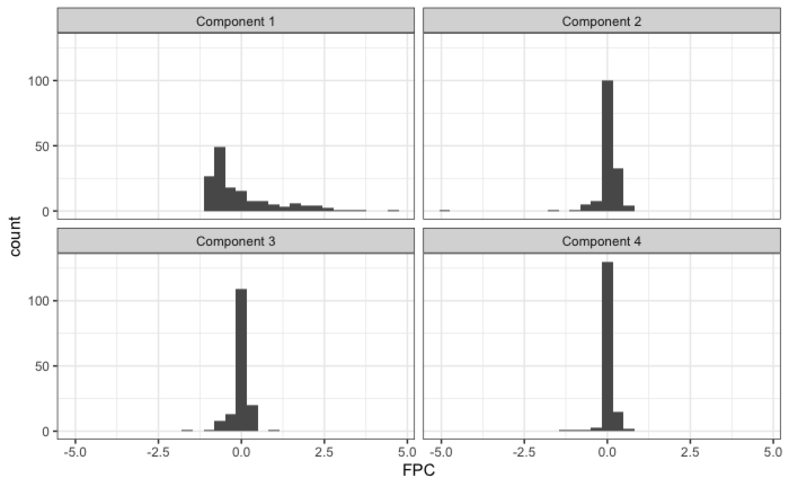

Supplement: S2 Fig — FPC scores were obtained by applying FPCA on the spatial entropy curves from the NSCLC dataset. The scores were centered around 0. (TIF) [file pcbi.1011490.s003.tif]

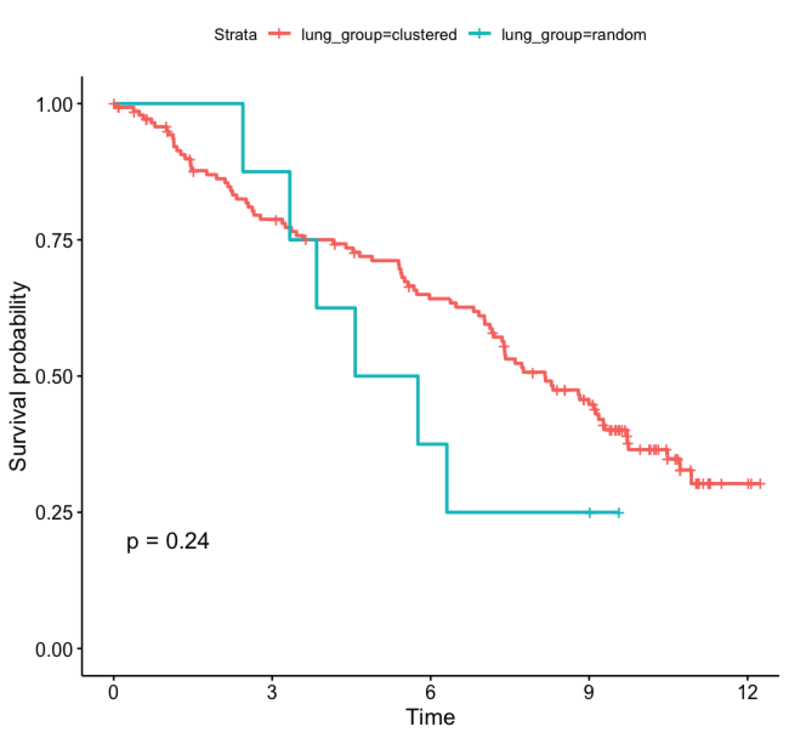

Supplement: S3 Fig — Subjects were classified as clustered vs. random based on the permutation test of the Mantel correlation. P-value of 0.24 indicates non-significant difference in survival probability in two groups. (TIF) [file pcbi.1011490.s004.tif]

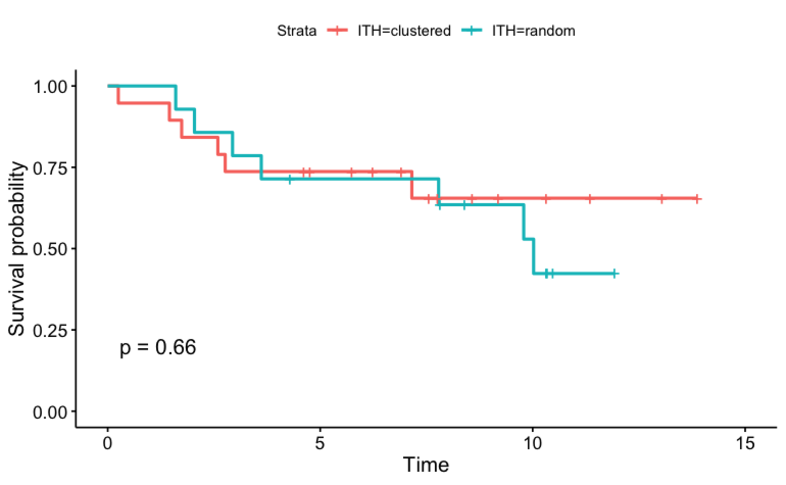

Supplement: S4 Fig — Subjects were classified as clustered vs. random based on the permutation test of the Mantel correlation. P-value of 0.66 indicates non-significant difference in survival probability in two groups. (TIF) [file pcbi.1011490.s005.tif]

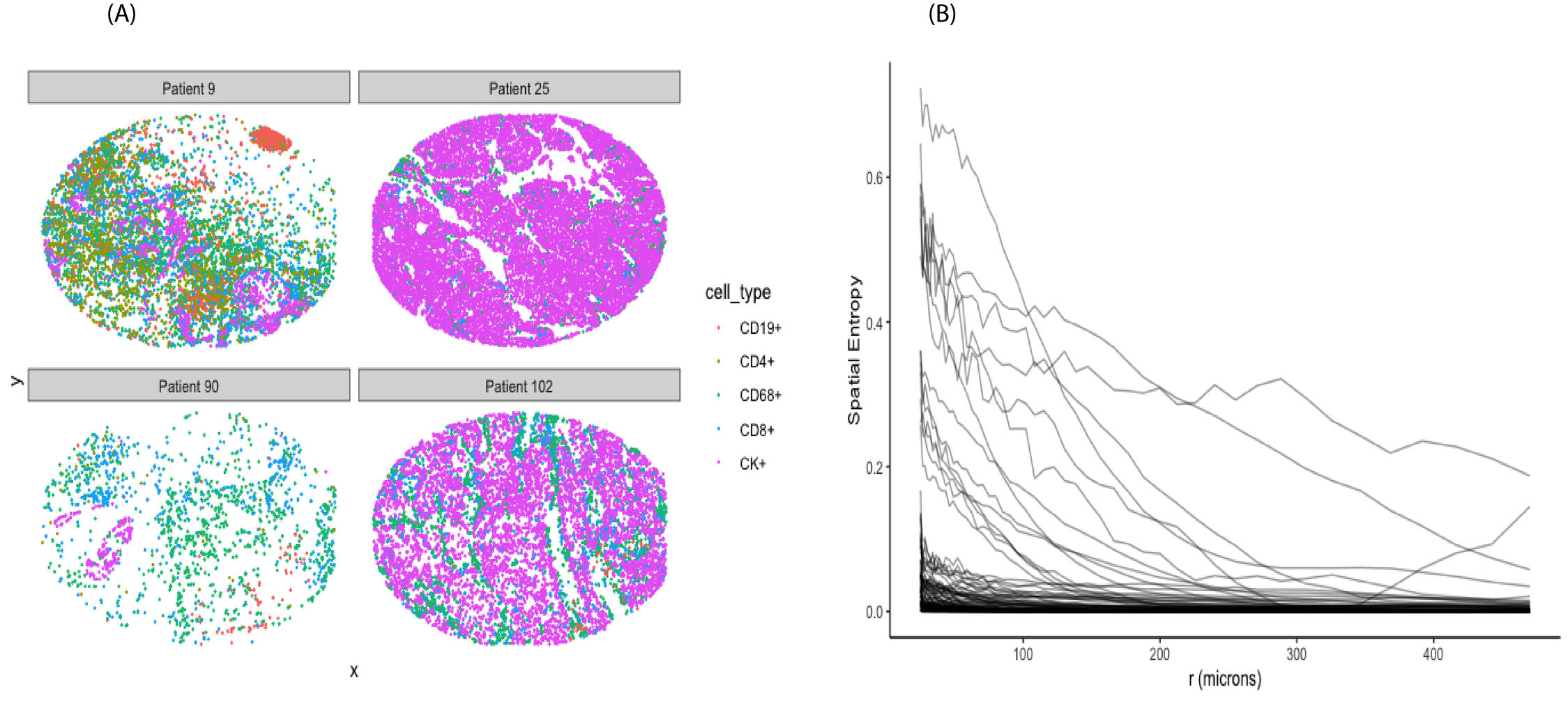

Supplement: S5 Fig — (A) Representative images with distribution of immune cells including CD19+ B cells, CD4+ T cells, CD8+ T cells, CD68+ macrophages, and CK+. (B) Spatial entropy of the five cell types as a function of inter-cell distances. (TIF) [file pcbi.1011490.s006.tif]

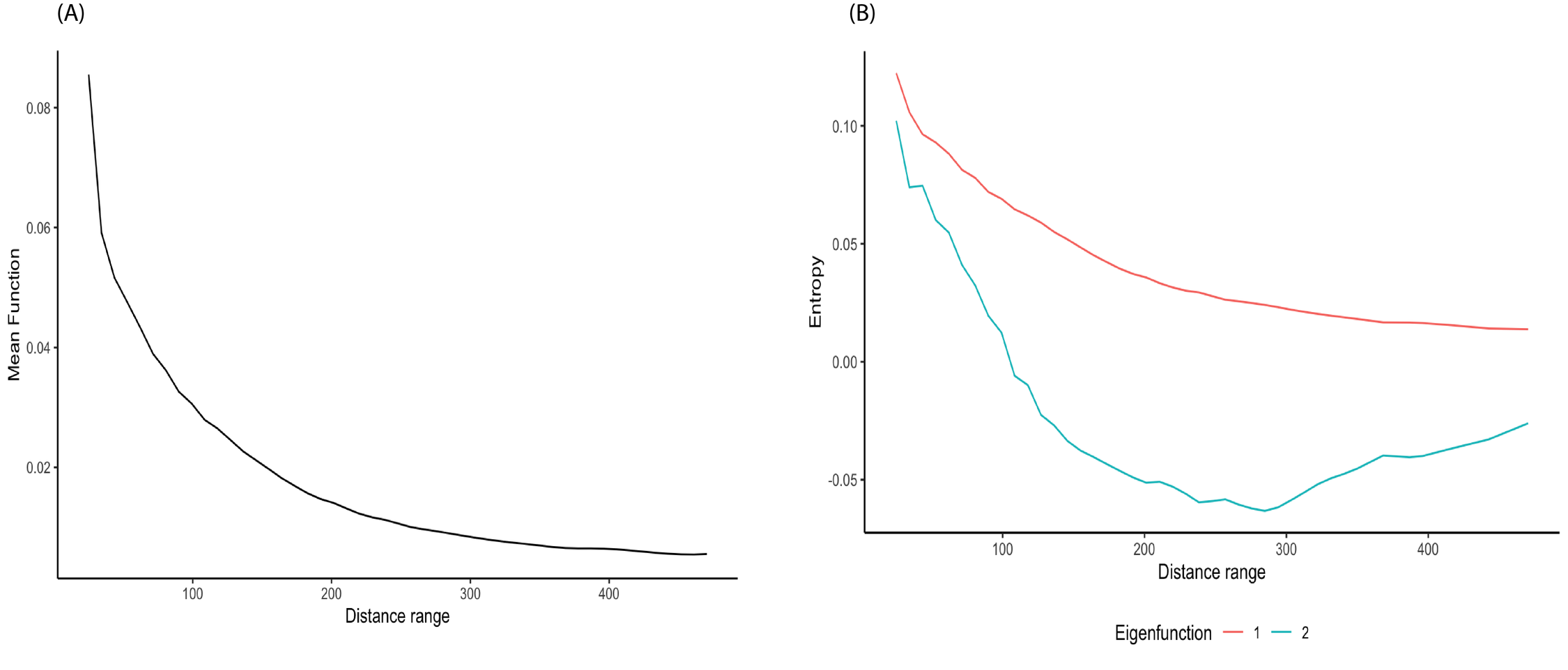

Supplement: S6 Fig — (A) Mean function. (B) First three eigenfunctions. (TIF) [file pcbi.1011490.s007.tif]

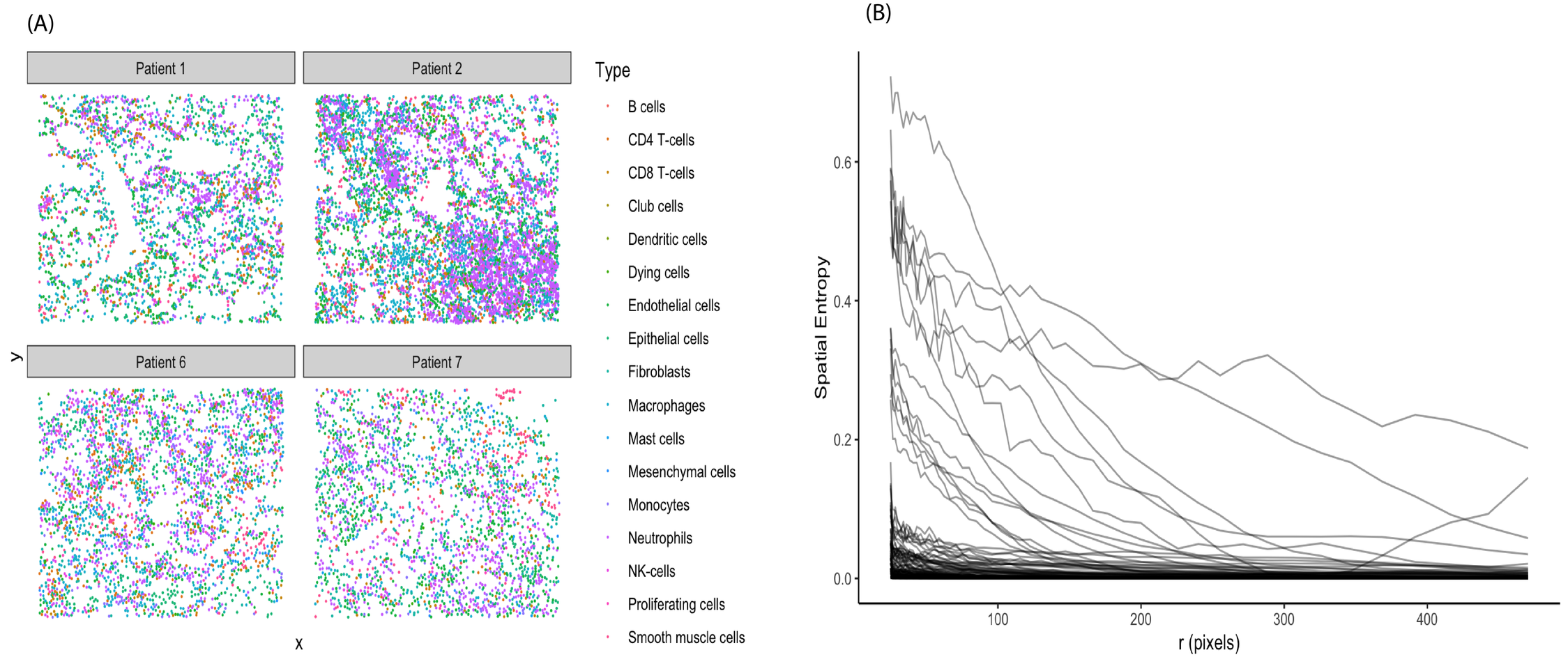

Supplement: S7 Fig — (A) Representative images with distribution of 17 different cell types (e.g., B cells, CD4 T cells, etc.) (B) Spatial entropy of all cell types as a function of inter-cell distances. (TIF) [file pcbi.1011490.s008.tif]

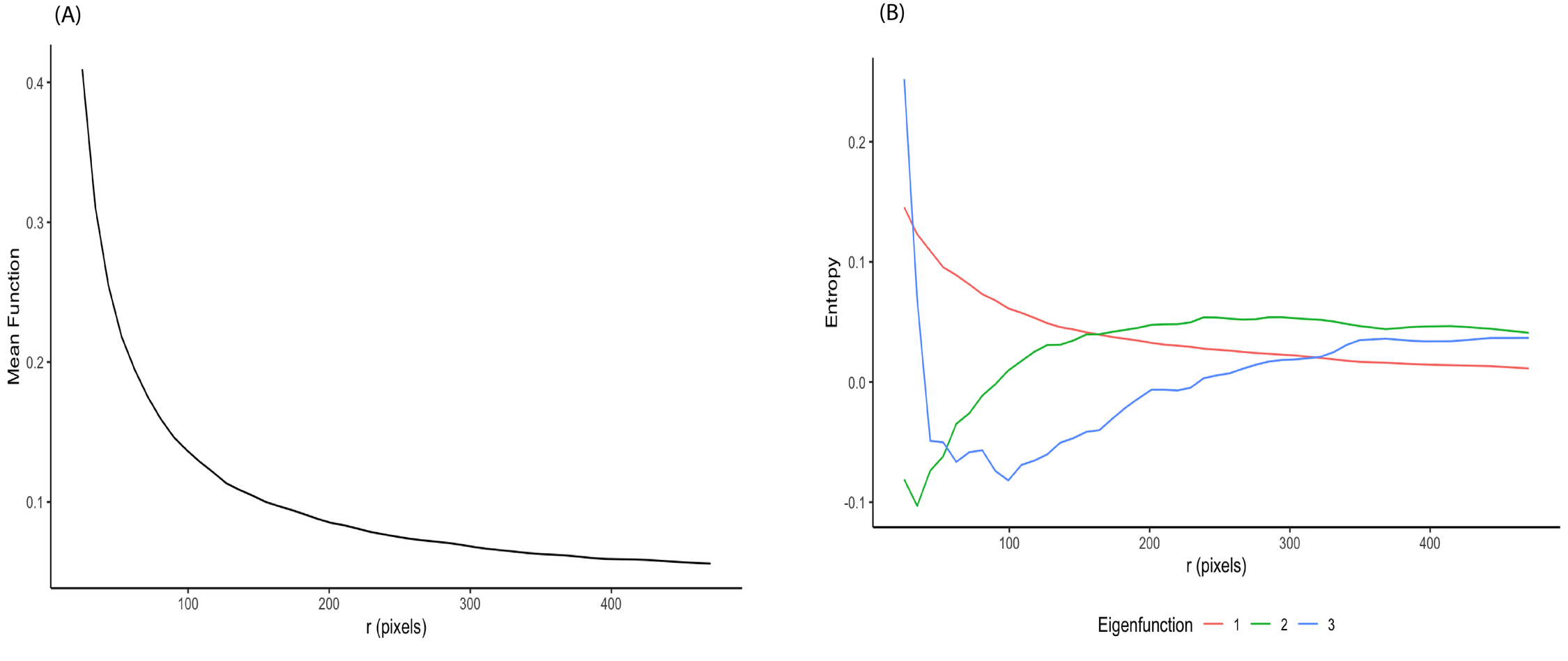

Supplement: S8 Fig — (A) Mean function. (B) First two eigenfunctions. (TIF) [file pcbi.1011490.s009.tif]

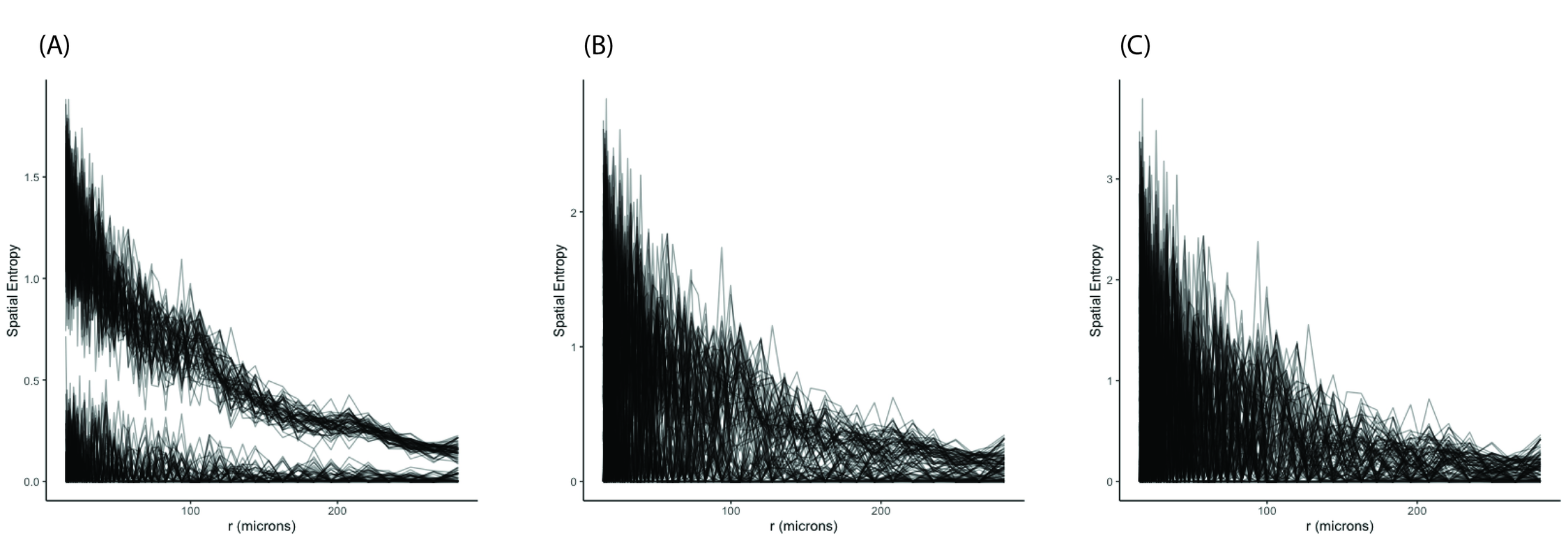

Supplement: S9 Fig — (A) low additive noise. (B) medium additive noise. (C) large additive noise. Three levels of noise were added to the reference SPI curves (clustered vs. random) to generate subject-specific SPI curves. (TIF) [file pcbi.1011490.s010.tif]

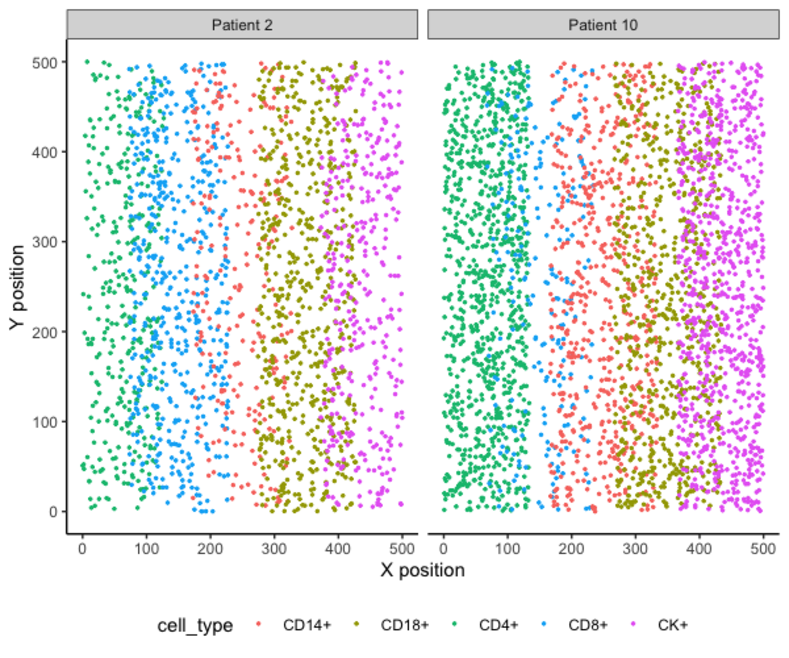

Supplement: S10 Fig — The number of cells for each cell type was simulated from a negative binomial distribution with the mean and dispersion parameter randomly selected from two ranges [200:500] and [1:3], respectively. The x-coordinates of some randomly selected cell were varied to create overlapping between clusters of cell types. (TIF) [file pcbi.1011490.s011.tif]

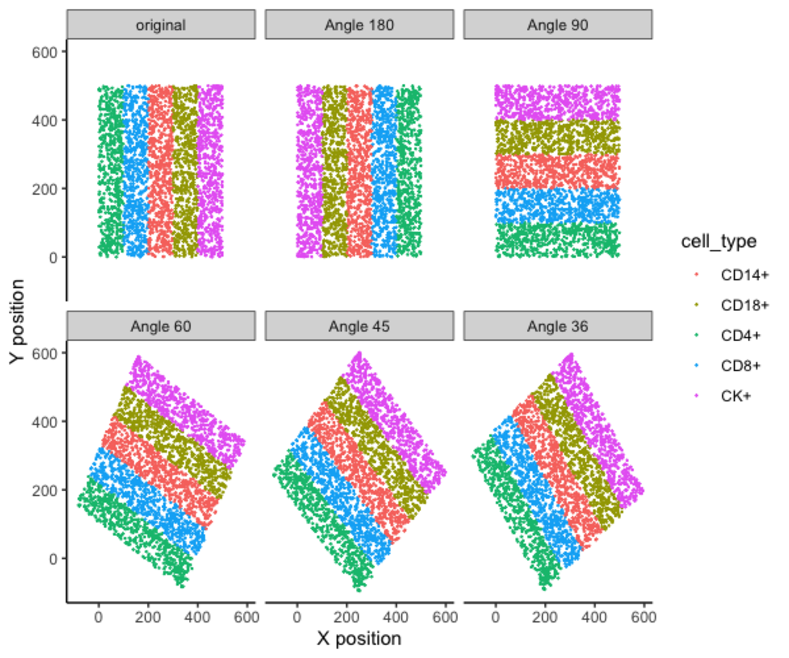

Supplement: S11 Fig — The simulated clustered point pattern (i.e., original) introduced in Fig 6A was rotated at various angles including 180°, 90°, 60°, 45°, and 36°. (TIF) [file pcbi.1011490.s012.tif]

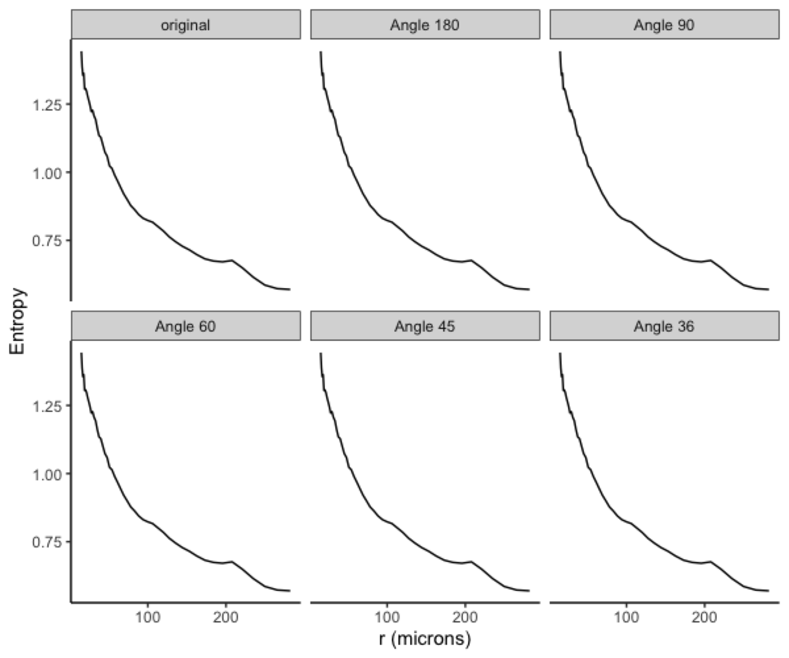

Supplement: S12 Fig — At each rotation, pairwise distances between all cells were calculated based on their corresponding x- and y-coordinates. Distance ranges were subsequently computed. Finally, the spatial entropy curve across all distance ranges was obtained. (TIF) [file pcbi.1011490.s013.tif]

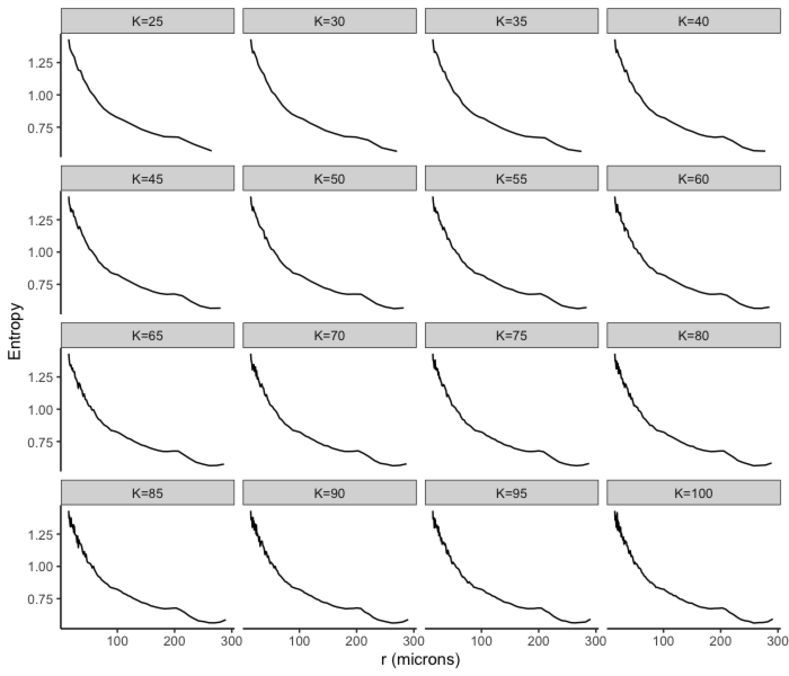

Supplement: S13 Fig — K values were incremented from 25 to 100 in steps of 5. At each K, a sequence of distance breaks was generated by linearly decreasing from dK to 0 on a log scale. Distance ranges were subsequently computed. Finally, the spatial entropy curve across all distance ranges was obtained. (TIF) [file pcbi.1011490.s014.tif]

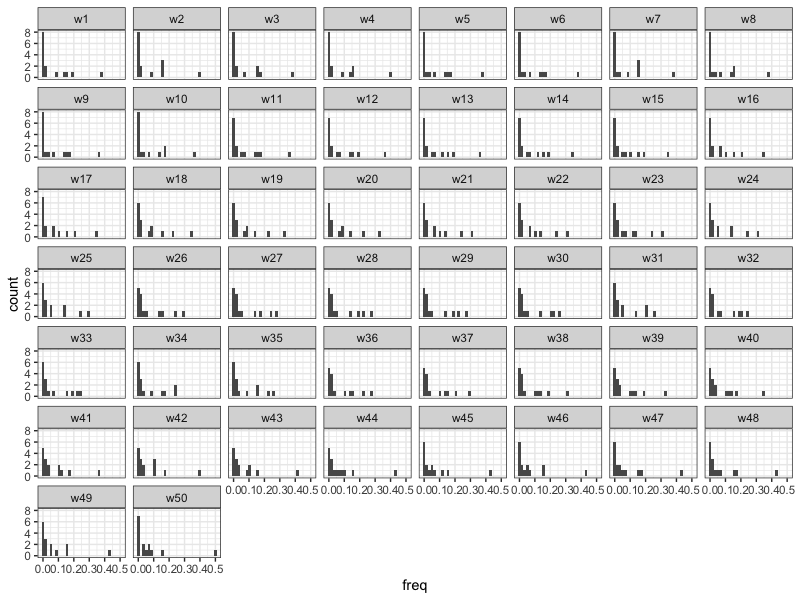

Supplement: S14 Fig — One representative image in the NSCLC dataset was used for demonstration. Based on pairwise distances between cells, we generated K = 50 distance breaks wk for k = 1, …, K. At each wk, the co-occurrences between cell types p(zr|wk) were obtained. We then built this panel of histograms using the relative frequencies of the cell type co-occurrences. (TIF) [file pcbi.1011490.s015.tif]

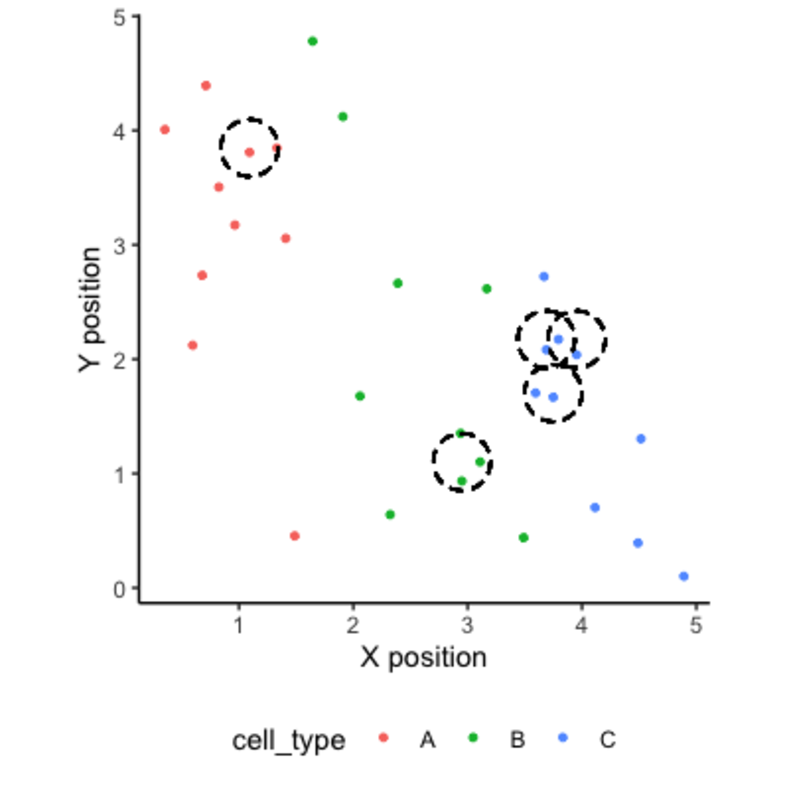

Supplement: S15 Fig — A point pattern consisting of 30 cells, with 10 cells per each type A, B, and C, was simulated. Circles of radius 0.25 were drawn around each point to identify co-occurrences of cell types with the first distance range w1 = (0, 0.25]. Specifically, there were 1 AA, 1 BB, 3 CC, 0 AB, 0 AC, and 0 BC. (TIF) [file pcbi.1011490.s016.tif]
