# Supplementary material for: FunSpace: A functional and spatial analytic approach to cell imaging data using entropy measures
Source: PLoS Comput Biol. 2023 Sep 27;19(9):e1011490. doi: 10.1371/journal.pcbi.1011490 (PMC10561868; doi:10.1371/journal.pcbi.1011490)
Supplement: Attachment — Submitted filename: FunSpace_ResponseLetter.pdf [file pcbi.1011490.s018.pdf]

Dear Dr. Patrick,

Thank you very much for reviewing our manuscript entitled: "FunSpace: A functional and spatial analytic approach to cell imaging data using entropy measures (PCOMPBIOL-D-22-01701). We are pleased to submit our revised paper for publication in PLOS Computational Biology. Two versions of the revised paper are included: one with changes highlighted in yellow and a clean version.

We appreciate the reviewers' constructive comments and suggestions, which led to a significant improvement of our paper. To facilitate the review process, in our letter, we provide a point-by-point response to each of the comments and mention the changes made to the original manuscript. We summarize the major changes below:

- In Sections S.4 and S.5 of the Supplementary Information, we have included additional analyses using two real datasets including ovarian cancer data (as part of the Bioconductor ExperimentHub package VectraPolarisData [1]) and an immunohistochemistry (IHC) data on lung tissues [2] during COVID-19 progression available at `https://doi.org/10.5281/zenodo.4633905`.
- In Sections S.6.2 and S.6.3 of the Supplementary Information, we have included two additional simulation studies to specifically assess the performance of the proposed framework in comparison to the model that incorporates Shannon entropy as a predictor.
- We have added two sensitivity analyses in Sections S.7.1 and S.7.2 in the Supplementary Information to explore the impact of rotation and varying the total number of distance breaks K on the resulting spatial entropy.
- We have accordingly revised the notations in the Model section based on the reviewers' suggestions.
- We have integrated all the functionalities of the proposed method into the `R` package `mxfda`, which can be found at `https://github.com/julia-wrobel/mxfda`. We are currently in the process of preparing its submission to CRAN.
- We have enhanced the quality of all figures in the revised manuscript.

Thank you very much for your time and consideration. We look forward to hearing from you.

Sincerely,

Thao Vu

Responses to Editor

We sincerely thank you for providing us constructive suggestions, which led to a significant improvement of the paper. Below are our responses to your comments which we reiterate in italics for your convenience.

1. *I strongly recommend that you make functions to run FUNSPACE available on CRAN or Bioconductor to ensure it can be used easily by the community.*

   Our response: Following your suggestion, functions accompanying FunSpace have been incorporated into the `R` package `mxfda`, which can be found at https://github.com/julia-wrobel/mxfda. We are currently in the process of preparing its submission to CRAN.

2. *Given the description on the github page, I would request that you clarify that this work is not concurrently under consideration at Bioinformatics.*

   Our response: We have removed the term "Bioinformatics" and accordingly modified introduction on Github as: "This repository contains code and scripts to accompany an article "FunSpace: A functional and spatial analytic approach to cell imaging data using entropy measures"."

3. *Your figures have rendered skewed and of varying quality in the submission process. You should double check this.*

   Our response: We have enhanced the quality of all figures in the revised manuscript.

Responses to Reviewer 1

We sincerely thank you for providing us constructive suggestions, which led to a significant improvement of the paper. Below are our responses to your comments which we reiterate in italics for your convenience.

Major comments

1. *One of the major claims in the paper is that the spatial entropy measures proposed in this manuscript can better describe the cellular architectures than other metrics. The author only demonstrated this point theoretically. However, no comparison was conducted in the downstream simulations or real data applications. Such comparison is necessary to tell if the spatial entropy is better than other existing measures in practice. For example, in the application to the NSCLC dataset, it is not surprising to find that the spatial heterogeneity described by the spatial entropy has a significant association with overall survival. I wonder if we use other metrics, e.g., the Shannon entropy, instead of the spatial entropy, and perform the same downstream analysis, we will arrive at the same conclusion. More comprehensive comparisons are desired to systematically show the advantage of spatial entropy over others. In the simulation and real data analysis, the authors can replace the spatial entropy with Shannon entropy, conduct the rest analysis, and compare the results with the current ones achieved using spatial entropy.*

   Our response: Following your input, we have included two additional simulation studies in Sections S.6.2 and S.6.3 of the Supplementary Information to specifically assess the performance of the proposed framework compared to the model that incorporates Shannon entropy as a predictor, as follows.

   Additional simulation 1: We computed reference Shannon entropy values [3] with respect to the two simulation spatial configurations in Fig. 6. To generate subject-specific Shannon entropy for each simulated image, we introduced three levels of additive noise (small, medium, and large) following Gaussian distributions with mean 0 and standard deviations of 0.25, 0.5, and 1, respectively. At each level of noise, we generated datasets of different sizes (N = 100, 200, 500, and 1000). Following the simulation in Section 4, we partitioned each dataset into training (75%) and testing (25%) sets. Four models were fit using the training set:(1) a

model accounting for both clinical predictor and spatial heterogeneity (2) a model accounting for only spatial heterogeneity, (3) a model accounting for only clinical predictor, and (4) a model accounting for spatial heterogeneity using Shannon entropy. We evaluated the predictive performance of the four different models on the testing sets using normalized root mean squared error (NRMSE). We repeated the simulation for 100 iterations and recorded the average NRMSE for each of the four models across four sample sizes: N = 100, 200, 500, 1000 in Table S1. The variation of NRMSEs decreased as sample sizes increased from N = 100 to N = 1000. Note that when there was a low level of subject-specific variation (Fig. S9 (a)), the separation between the two spatial configurations was clear. As a result, all four models performed almost equivalently, particularly at the largest sample size N = 1000. However, when the additive noise was greatly increased, the difference in the spatial entropy curves across the two patterns was not as pronounced (Fig. S9 (c)). In the extreme case at N = 100, by accounting for the impact of the heterogeneity in the spatial configurations, models (1) and (2) yielded significantly smaller NRMSEs compared to the remaining two models. Without accounting for the spatial information, model (4) that used Shannon entropy as a predictor, exhibited relatively higher NRMSEs. However, as the sample size increased, the benefit of accounting for spatial impact became less noticeable. In other words, there was no significant difference in the performance of the four models.

Additional simulation 2: Using a similar simulation setup, we assumed that there were two groups of subjects. The number of subjects per group followed a binomial distribution with a probability of 0.5, i.e., $N_g \sim Binom(N = 100, 0.5)$ with $g = 1, 2$. We considered two spatial configurations: clustered vs. random (Fig. 6 a,b), corresponding to $g = 1, 2$, respectively. However, rather than generating spatial entropy curves specific to each subject by adding noise to the reference curves, we directly simulated subject-specific point patterns. Specifically, we simulated the number of cells for each cell type: CD14+, CD19+, CD4+, CD8+, and CK+, from a negative binomial distribution with the mean and dispersion parameter randomly selected from two ranges [200:500] and [1:3], respectively. Next, based on the previous clustered configuration (Fig. 6a), we varied the x-coordinates of some randomly selected cells to create overlapping between clusters of cell types. Fig. S10 shows two representative simulated images. The rationale behind this setup was to introduce variations in both the total number of cells per cell type and the spatial information. We then computed spatial entropy curve and Shannon entropy for each simulated image. Following the proce-

dure described above, the dataset was partitioned into training (75%) and testing (25%) sets. We fit four models on the training set: (1) a model accounting for both clinical predictor and spatial heterogeneity (2) a model accounting for only spatial heterogeneity, (3) a model accounting for only clinical predictor, and (4) a model accounting for spatial heterogeneity using Shannon entropy. Given the computational intensity, we performed 45 simulations at N = 100 and recorded the NRMSE distribution for each model in Table S2. As expected, model (1) that accounted for both clinical effect and spatial heterogeneity, exhibited significantly smaller NRMSEs than the other three models. Notably, model (4) which incorporated Shannon entropy to capture cell type diversity but not their relative spatial distribution, resulted in the largest NRMSEs.

Real data analysis: We previously compared the proposed approach using the first few FPCs of spatial entropy with four other diversity metrics including Shannon entropy [4], Simpson entropy [4], ecosystem diversity index (EDI) [5], and Mantel correlation [6] with regard to identifying a significant association between the spatial heterogeneity in the TME and overall survival. We included all the model outputs for the NSCLC and TNBC datasets in Sections S.2 and S.3 of Supplementary Information, respectively.

2. *Although the author claims that FunSpace can be used to study various tumor types, the current evaluation is only based on lung and breast cancer data. It is unclear how generalizable this method is. It appears various tuning and adjustments of some parameters are needed. The idea may work well for lung and breast cancer data, but may not be readily usable in other cancer types.*

   Our response: In response to your suggestion, we have added analyses for two other datasets including ovarian cancer data (as part of the Bioconductor ExperimentHub package VectraPolarisData [1]) and an immunohistochemistry (IHC) data on lung tissues [2] during COVID-19 progression available at https://doi.org/10.5281/zenodo.4633905 in Sections S.4 and S.5 of the Supplementary Information. Specifically, in the ovarian cancer dataset, we investigated the association between spatial heterogeneity in the ovarian tumor microenvironment with overall survival. In the COVID-19 progression data, we used spatial heterogeneity to build a model classifying 23 patients with different stages of acute lung injury including healthy, acute respiratory distress syndrome (ARDS), pneumonia, influenza, early COVID-19, and late COVID-19.

3. *Following my previous comments, some critical parameters' values are determined arbitrarily, which limits the usefulness of FunSpace in practice. For example, the distance breaks d_k are critical in the calculation of spatial entropy. The author states that they can "be flexibly chosen depending on the specific applications" (line 117). How the choice of these parameters affects the downstream results? How to choose the value of these parameters when analyzing different tumor data? Some guidance is in demand.*

   Our response: We have added a sensitivity analysis in Section S.7.2 to explore the impact of varying the total number of distance breaks, denoted by K, on the resulting spatial entropy curves. Particularly, we utilized the simulated clustered configuration depicted in Fig. 6a as a case study. We computed the pairwise Euclidean distances between all points in the pattern based on their xy-coordinates. As the general focus was on local cell-to-cell interactions, we specifically considered short pairwise distances. We set the maximum distance break for consideration d_K to be the median of the distance distribution.

   We then generated a sequence of distance breaks of length K by linearly decreasing from d_K to 0 on a log scale. Fig. S13 shows the spatial entropy curves corresponding to different K values which were incremented from 25 to 100 in steps of 5. As K decreased, the distance ranges expanded, leading to a higher number of co-occurrences between cell types within each range. This resulted in smoother spatial entropy curves. On the contrary, as K increased, the spatial entropy curves showed slightly more fluctuations at small distances. Nevertheless, the overall trend of the spatial entropy remains consistent across all values of K.

4. *The spatial entropy summarizes the cell type heterogeneity of the tumor region using only one value. It is an oversimplified representation of the complex tumor microenvironment, which have very limited interpretability. In both the application to lung and breast cancer datasets, the final conclusions are both "the association between the heterogeneity in spatial architectures of immune-tumor interactions and survival outcome is significant". It would be great if the author could provide more insight into this association. For example, which cell pair's distribution is more important in this association?*

   Our response: In the Discussion and Conclusions section, we have provided a brief description to reemphasize the novelty of this proposed approach. Unlike existing frameworks, including our recent SPF approach [7], which focus on spatial interactions between each pair of cell types individually, our new approach takes a more comprehensive approach by consid-

ering the spatial interactions between multiple cell types simultaneously. We have added the following paragraph to discuss some limitations of the proposed approach.

"While the approach does not provide a breakdown of which pairs of cell types driving the spatial heterogeneity due to its aggregated nature, it serves as an initial step in identifying specific distance intervals with significant variation to focus on. Subsequently, existing approaches such as the K-function can be employed to analyze specific pairs of cells of interest individually."

Minor comments

1. *Some improvements can be made to make the method section easier to read. For example, in Lin 116, the variable K is used without definition. In line 145, lowercase s is not defined.*

   Our response: We have revised the notations in those paragraphs as follows.

   "Denote $w_k = (d_{k-1}, d_k]$, with $k = 1, \ldots, K$ and K is the total number of distance breaks. Here, d_k is a fixed set of distance breaks which are a function of pairwise distances between all points in the pattern, referred to as inter-point distances. These distance breaks d_k can be flexibly chosen depending on the specific applications. In our context, as the general focus was on local cell-to-cell interactions, we specifically set the initial distance d_0 to 0 and d_K to be the median of the inter-cell distance distribution."

   "Assume that spatial entropy for subject i at distance break k, SPI_{ik}, is generated from the underlying smooth random function $X(s)$ at a random distance s, with known mean function $EX(s) = \mu(s)$ and covariance function $Cov(X(s), X(t)) = G(s,t)$. The domain of $X(.)$ typically is a bounded and closed distance interval $\mathcal{S}$."

2. *Since spatial entropy is a novel measure, it would be helpful to discuss its properties. For example, will different spatial configurations yield the same spatial entropy? Is the spatial entropy invariant under rotation?*

   Our response: Spatial entropy will vary across different spatial configurations as we demonstrated in Fig. 6 for the clustered vs. random configurations. However, when the spatial configuration is held constant, we expect that spatial entropy is invariant under rotation. This is because spatial entropy is computed based on pairwise distances across all the points in the pattern. In other words, the spatial entropy should remain unaffected even if the xy-

coordinates of the points are altered, as long as the relative distances between them remain consistent.

As a proof of concept, we rotated the clustered point pattern introduced in Fig. 6a at various angles to obverse any potential changes in the resulting spatial entropy. Fig. S11 shows the original point pattern and its rotations at 180°, 90°, 60°, 45°, and 36°. Fig. S12 displays the corresponding spatial entropy which remains unchanged across the five rotations.

Responses to Reviewer 2

We sincerely thank you for providing us constructive suggestions, which led to a significant improvement of the paper. Below are our responses to your comments which we reiterate in italics for your convenience.

Major comments

1. *In interpreting equation (1), the authors mentioned "In our context, the maximum entropy is reached when cells of different types are scattered evenly on a given image. ". I believe the authors meant same number of cells in every cell type? Because the in this equation the spatial information is not included*

   Our response: We have accordingly revised the sentence as follows.

   "In our context, the maximum entropy is reached when we have the same number of cells for all cell types in a given image"

2. *Is x_i a vector, and the element is 1 where cell is in cell type i and 0s everywhere else? Or is it an integer indicating the cell type of a cell (like 1, 2, 3, . . .)*

   Our response: We defined each x_i as an integer indicating a cell type.

3. *The definition of co-occurrence of cells is not fully clear to me - is it the possible combination of types of two cells (x_i and $x_{i'}$), or is it defined as the co-occurrence of two cell types within some region? I assume that it is the combination of cell types of any two cells in the image?*

   Our response: You are correct that we defined co-occurrences as all possible combinations of cell types of any two cells in an image. As an example, if cells in a particular image are phenotyped as A, B, and C, the corresponding co-occurrences would include AA, BB, CC, AB, AC, and BC. Additionally, when space was incorporated, we were able to empirically estimate the distribution of these co-occurrences at some region or distance range of interest, i.e., $p(z_r|w_k)$ in the paper.

4. *Can the authors justify the decomposition of $H(Z)$? And the definition if $SPI(Z)$? Is it different from $SPI_k(Z)$?*

   Our response: On page 7 of the revised manuscript, we have clarified $SPI(Z)$ as the overall spatial information, which can be computed as a weighted sum of partial spatial entropy at

each distance range w_k, denoted as $SPI(Z|w_k)$ or SPI_k. Additionally, we have included a detailed derivation for the decomposition of $H(Z)$.

5. *What is the empirical distribution of $p(z_r|w_k)$ in a real image?*

   Our response: We have added Fig. S14 in the Supplementary Information to show the empirical distribution of $p(z_r|w_k)$ for $k = 1, \ldots, 50$ using a representative image from the NSCLC dataset. By conditioning on the space variable W, the distributions are right-skewed across all distance breaks w_k, with a skew towards low co-occurring pairs.

6. *Can the authors justify using local smooth functions for SPI and give detailed steps of smoothing? Especially for X(s) and G.*

   Our response: We have included a clarification following Eq. (3) in the revised manuscript as follows.

   "We implemented the `R` package `fdapace` by Yao et al. [8] to estimate the mean function $\hat{\mu}(s)$ and the covariance surface $\hat{G}(.)$ based on the pooled data from all subjects. Details of the local linear smoothers for function and surface estimation are provided in Section S.1 of the Supplementary Information."

   We have added Section S.1 in the the Supplementary Information to highlight the detailed equations for estimating $\hat{\mu}(s)$ and $\hat{G}(.)$.

Minor comments

1. *i is cell type in method section but subject in model section*

   Our response: We have redefined the cell type variable as Y with the corresponding index from $j = 1, \ldots, J$. The index $i = 1, \ldots N$ has been retained to indicate subjects.

   "Let Y be a discrete random variable denoting a cell type, with the total of J possible types. Shannon entropy [3] is the expected value of an information function measuring the uncertainty in observing $Y = y_j, j = 1, \ldots, J$ with the corresponding probability mass function (pmf) as $p_Y = (p(y_1), p(y_2), \ldots p(y_J))^T$."

2. *same for the X notation, cell identification in method section, but a function in model section*
   3. and same for Sik

Our response: We have redefined the cell type variable as Y with the corresponding index from $j = 1, \ldots, J$. The index $i = 1, \ldots N$ has been retained to indicate subjects. We have denoted SPI_{ik} as the kth observation of the random function $X_i(.)$ at a random distance S_{ik}.

Responses to Reviewer 3

We sincerely thank you for providing us constructive suggestions, which led to a significant improvement of the paper. Below are our responses to your comments which we reiterate in italics for your convenience.

Major comments

1. *The paper states that the proposed approach accounts for the "simultaneous heterogeneity caused by both cellular diversity and spatial architecture of multiple cell types". This is an important statement through which the readers get to know what results the method can generate as well as how to interpret them. So, it would be good to elaborate on both points, especially the later one "spatial architecture" because spatial architecture can be interpreted in many ways and the author should point out that what type of spatial architecture FunSpace focuses on. Since the proposed entropy method is of a flavor of a summary statistics of the tissue slices and it won't be able to retain all the spatial architecture information in the raw data.*

   Our response: Initially, we used the term "spatial architecture" to describe the distribution of cells in space. However, based on your suggestion, we have replaced "spatial architecture" with "spatial distribution of cells" or "spatial organization of cells" throughout our revised manuscript. Furthermore, we have included the following sentences in the Introduction to elaborate on the novelty of the proposed approach, as follows.

   "..., we propose an approach that leverages spatial entropy measures [9] to account for the spatial heterogeneity of multiple cell types simultaneously across subjects at different distance ranges and how such variability impacts a clinical outcome of interest. More precisely, when incorporating space into entropy measures, the proposed framework is able to capture the diversity in cellular composition, such as similar proportions across cell types or dominance of a single type, at a specific distance range. Additionally, spatial patterns, including clustered, independent, or regular, among cell types can also be acquired."

2. *While introducing the proposed spatial entropy measures (X, Z, and W), it would be better to connect the random variables as well as other statistical concepts to the real data and provide corresponding (synthetic) examples with figures, so that readers who are not very familiar with*

either entropy theory or the spatial data can better understand the proposed idea. For example, give an example to show how the SPI is calculated with (synthetic) data and figures. Also, there are a couple of concepts brought up by the paper for example "inter-point distance" and "inter-cell distance" that can be unclear for readers who are not familiar with spatial data, and concepts like "observation windows" that can be unclear for general reads. It would be good to introduce and define these concepts so that the proposed method can be more easily accepted and better appreciate by broader audiences.

Our response: We have revised the paragraph following Eq.(2) to remove the term "observation window" and provide a clearer definition of "inter-point distances", as follows.

"Denote $w_k = (d_{k-1}, d_k]$, with $k = 1, \ldots, K$ and K is the total number of distance breaks. Here, d_k is a fixed set of distance breaks which are a function of pairwise distances between all points in the pattern, referred to as inter-point distances."

Furthermore, we have redefined Y as a random variable denoting a cell type in Section 2.1.1 of the revised manuscript. We have added Section S.8 in the Supplementary Information to illustrate the spatial entropy computation using the random variables Y , Z, and W that were defined in the main paper. To accomplish this, we simulated a point pattern (Fig. S15) consisting of 30 cells, with 10 cells per each type A, B, and C, which were represented by the Y random variable. Then, we defined co-occurrences (denoted by Z) as all possible pairs of cell types, including AA, BB, CC, AB, AC, and BC. Based on the pairwise distances between all cells in the pattern, we generated distance breaks d_k for $k = 1, \ldots, K$ of length $K = 3$. Consequently, we had three contiguous distance ranges (i.e., W random variable) of $w_1 = (0, 0.25]$, $w_2 = (0.25, 1.12]$, and $w_3 = (1.12, 7]$. We have provided detailed equations for calculating the spatial entropy at the first range, $w_1 = (0, 0.25]$, denoted by $SPI_1 = SPI(Z|w_1)$.

Minor comments

1. *Line 95. How does X (as a random variable) model the probability of cell types? Does the paper assume the cell types of all cells are iid as X?*

   Our response: We have redefined Y as a random variable denoting a cell type with the probability mass function $p_Y = P(Y = y_j)$ for $j = 1, \ldots, J$ in Section 2.1 of the revised

manuscript. Then, the entropy $H(Y) = \sum_{j=1}^{J} p(y_j) \log\left(\frac{1}{p(y_j)}\right)$ ranges between 0 and $\log(J)$; and its maximum is achieved when Y is uniformly distributed.

2. *Fig1 lower right subfigure. The curve of small r fluctuates up and down a little bit and is not smooth. Is this pattern commonly observed in real data? Should this affect the choice of distance intervals? Do we need to pre-smooth the curve before using FPCA?*

   Our response: We generated Fig. 1 using two representative images from the NSCLC dataset. You are correct that the spatial entropy curve exhibited slight fluctuations at short distances. This is not unexpected since we intentionally focused on small distance intervals to explore local cell-to-cell interactions. As the distance ranges became narrower, there was increased variation in the observed number of co-occurrences between cell types.

   We have included a sensitivity analysis in Section S.7.2 of the Supplementary Information to explore the impact of varying the total number of distance breaks, denoted by K, on the resulting spatial entropy curves. As expected, we observed smoother spatial entropy curves when K decreased (i.e., larger distance intervals). When K increased, the spatial entropy curves showed slightly more fluctuations at small distances. Nevertheless, the overall trend of the spatial entropy remains consistent across all values of K. Provided that FPCA [8] applied local linear smoothers to estimate the mean function and covariance surface, we did not need to pre-smooth the spatial entropy curves.

3. *Line 104 and 109. How does the "co-occurrence of cells" explicitly define? Do the cells with cell type x_i and the cell with cell type $x_{i'}$ need to be spatially close to each other so as to be counted as an instance of co-occurrence of cell types x_i and $x_{i'}$?*

   Our response: We defined co-occurrences as all possible combinations of cell types of any two cells in an image. As an example, if cells in a particular image are phenotyped as A, B, and C, the corresponding co-occurrences would include AA, BB, CC, AB, AC, and BC. Additionally, when space was incorporated, we were able to empirically estimate the distribution of these co-occurrences at some region or distance range of interest, i.e., $p(z_r|w_k)$ in the paper.

4. *Line 114. Better to define "points" and "observation windows" here.*

   Our response: To enhance clarity, we have removed the term "observation window" and replaced "points" with "cells".

5. *Line 121. Better to introduce more about what do "entropy due to space" and "remaining heterogeneity" mean, possibly with real data or synthetic data examples with figures.*

   Our response: We have included Section S.8 in the Supplementary Information to illustrate the computation of spatial entropy (i.e., entropy due to space) and residual entropy (i.e., remaining heterogeneity) at three distance intervals w_1, w_2, and w_3, using simulated data.

6. *Line 129. When having more than two cell types, if a large SPI at a distance is observed, how to figure out which pair of cell types (mostly) contributed to the heterogeneity?*

   Our response: In the Discussion and Conclusions section, we have provided a brief description to reemphasize the novelty of this proposed approach. Unlike existing frameworks, including our recent SPF approach [7], which focus on spatial interactions between each pair of cell types individually, our new approach takes a more comprehensive approach by considering the spatial interactions between multiple cell types simultaneously. We have added the following paragraph to discuss some limitations of the proposed approach.

   "While the approach does not provide a breakdown of which pairs of cell types drive the spatial heterogeneity due to its aggregated nature, it serves as an initial step in identifying specific distance radii with significant variation to focus on. Subsequently, existing approaches such as the K-function can be employed to analyze specific pairs of cells of interest individually."

7. *Line 130. The heading of Section 2.2 is "Model". Therefore, is Section 2.1 not part of the model? Maybe consider better section headings so that readers can aware both sections are blocks of the proposed method/model.*

   Our response: Under the Materials and Methods section, we have updated the heading for Section 2.1 as "Model" to include three subsections: 2.1.1 for "Spatial entropy measures", 2.1.2 for "Functional principal component analysis (FPCA), and 2.1.3 for "Cox regression model".

8. *Line 137, Would* $K_i < 50$ *always work well? If not, may also give some suggestions and criteria in case users need to choose* K_i *by themselves for their own data. And what does "distance range" specifically and explicitly refers to here?*

   Our response: We have added a sensitivity analysis in Section S.7.2 to explore the impact of varying the total number of distance breaks, denoted by K, on the resulting spatial entropy curves. Particularly, we utilized the simulated clustered configuration depicted in Fig. 6a as a

case study. We computed the pairwise Euclidean distances between all points in the pattern based on their xy-coordinates. As the general focus was on local cell-to-cell interactions, we specifically considered short pairwise distances. We set the maximum distance break for consideration d_K to be the median of the distance distribution.

We then generated a sequence of distance breaks of length K by linearly decreasing from d_K to 0 on a log scale. Distance range was denoted as $w_k = (d_{k-1}, d_k]$, with $k = 1, \ldots, K$. Fig. S13 shows the spatial entropy curves corresponding to different K values which were incremented from 25 to 100 in steps of 5. As K decreased, the distance ranges expanded, leading to a higher number of co-occurrences between cell types within each range. This resulted in smoother spatial entropy curves. On the contrary, as K increased, the spatial entropy curves showed slightly more fluctuations at small distances. Nevertheless, the overall trend of the spatial entropy remains consistent across all values of K.

9. *Line 138. It would be better to numbering the subfigures instead of describing them with top/bottom row/column.*

   Our response: We have accordingly labeled individual panels of all figures in the revised manuscript.

10. *Line 140. There are existing methods for modeling functional covariates. The paper can talk about the benefits of using FPCA in the proposed approach.*

    Our response: In Section 2.2.1, we have discussed the advantage of utilizing FPCA in the proposed framework, as follows.

    "Since the distance ranges may vary across subjects depending on the relative distances between cells, the resulting spatial entropy functions SPI_i can be irregularly observed. Modeling these SPI directly as functional covariates as in Vu et al. [7] is possible in principle but would require methodological developments that are beyond our scope. Instead, we perform functional principal components analysis (FPCA) to obtain functional principal component (FPC) scores that can be utilized as scalar covariates in a standard modeling framework [8]."

11. *Line 144. Consider better introducing the "spatial entropy for subject i at distance break k" here, possibly with examples. And may also mention distance r here, since r is the distance that will be used for plotting and interpretation later.*

    Our response: We have modified the first paragraph of Section 2.2.1 as follows.

"Our goal is to use entropy measures defined in Section 2.1 for modeling patient-level outcomes. For each i-th subject, we have a collection of cells with their corresponding xy-coordinates available. We first define distance breaks and ranges for each i-th subject as d_{ik} and w_{ik}, respectively, such that $w_{ik} = (d_{i(k-1)}, d_{ik}]$ for $k = 1, \ldots, K_i$ and $i = 1, \ldots, N$. Spatial entropy for the i-th subject is represented as $SPI_i = \{SPI_{ik}\}_{k=1}^{K_i} = \{SPI_i(Z|w_k)\}_{k=1}^{K_i}$."

Additionally, we have provided a detailed illustration for calculating spatial entropy at different distance ranges SPI_{ik} using simulated data in Section S.8 of the Supplementary Information .

Finally, to maintain consistency in notation, we have replaced the distance label on the x-axis of all figures in the revised manuscript from r (microns) to d (microns).

12. *Line 145. Does FPCA requires the data to fulfill certain assumptions? If yes, do the data (entropy curves) satisfy these assumptions? And is X here the same or similar random variable as the X used in around line 97? If not, may consider change to another notation to better differentiated these two objects.*

    Our response: As mentioned in Section 2.2.1, we assumed the FPC scores ξ_l to be uncorrelated random variables with mean 0 and variance $E\xi_l^2 = \lambda_l$ with $\sum_l \lambda_l < \infty$, $\lambda_1 \geq \lambda_2 \geq \ldots$. We have included histograms for the first four FPC scores obtained from the NSCLC dataset in Fig. S2 of Section S.1 of the Supplementary Information. These scores were centered around 0, indicating that they satisfied the model assumptions.

    To clarify the notations, we have redefined Y (previously referred to as X in line 97) as a random variable denoting a cell type with the probability mass function $p_Y = P(Y = y_j)$ for $j = 1, \ldots, J$ in Section 2.1 of the revised manuscript. We have retained $X(.)$ to denote a random function from which the spatial entropy curves were generated.

13. *Line 152. To pool X(s) from multiple subjects, do the paper require the slices from different subjects have similar size and resolution? Do they need to come from the same technology with the same (or similar) cell type composition(s)?*

    Our response: Your observation is accurate regarding the importance of similar resolution across images and a common set of cell types when pooling information from multiple subjects. For instance, in the NSCLC dataset, all images were collected at a single-cell resolution using the Vectra 3.0 microscope (Akoya Biosystems), ensuring that they had similar ranges

of distance between cells. Moreover, as the samples were stained with the same panel of antibodies, the acquired images also shared a common set of cell types, including CD14+, CD19+, CD4+, CD8+, and CK+.

We have clarified this point in Section S1 of the Supplementary Information as follows.

"We directly implemented the `R` package `fdapace` by Yao et al. [8] to estimate the mean function $\hat{\mu}(s)$ and the covariance surface $\hat{G}(.)$ based on pooled data from all subjects. It is important to note that in the context of imaging data, the images should be collected at the same resolution so that ranges of distance between cells are similar. Furthermore, the images should also share a common set of cell types to ensure a comparable scale of entropy. Briefly, the authors defined the local linear scatterplot smoother for $\mu(s)$ by minimizing..."

14. *Line 160. How does the FPC scores look like? Are the scores corresponding to different FPCs have similar scales? If not, do we need to process the FPC scores before applying the Cox model?*

    Our response: We have included histograms for the first four FPC scores obtained from the NSCLC dataset in Fig. S2 of Section S.1 of the Supplementary Information. These scores have mean 0 and variance $E\xi_l^2 = \lambda_l$ with $\sum_l \lambda_l < \infty$, $\lambda_1 \geq \lambda_2 \geq \ldots$. Since they all captured the spatial entropy, it was not necessary for us to scale them prior to fitting the Cox regression model.

15. *Line 170. FPC scores accounting for 96% variance is included in the Cox model. How does the later FPCs, for example the 10th, 50th, 100th, look like? Are their patterns informative?*

    Our response: We have included Fig. S1 in Section S.1 of the Supplementary Information overlaying, for each of the five leading FPCs, the estimated mean function +/- each FPC score multiplying two standard deviations (SDs) of the associated score distribution. It is not surprising that the first two FPCs present more deviation from the mean as they account for 78.4% and 14.8% of total variance, respectively, while the remaining FPCs do not express informative patterns.

16. *Line 181. It seems the x/y coordinates, marker expression, and cell phenotypes are all columns of the matrix. Then, is the dimension of the matrix more than 2?*

    Our response: You were correct that the matrix had more than two dimensions. We have clarified this in the revised manuscript as follows.

"...to obtain a data matrix with rows corresponding to individual cells and multiple columns corresponding to x- and y-coordinates of each cell on the image, individual marker expressions, and cell phenotypes."

17. *Line 208. It seems only the first two FPCs are plotted instead of 3.*

    Our response: We have fixed this typo in the revised manuscript, as follows.

    "Fig. 3 shows the estimated mean function (A) and the first two eigenfunctions (B)."

18. *Line 227. This data has in total 36 antibodies available. Is it possible to analyze these 36 markers? If yes, how the results look like compared to the current results with I=2 (immune and tumor cell types)?*

    Our response: In Section 2.2.2 of the revised manuscript, we have clarified that the 36 markers were used to classify each individual cell as immune, stromal, tumor, and so on. Based on the cell types, we computed spatial entropy curves and subsequently investigated their association with survival outcomes using our proposed framework. Inspired by the findings of the original study [10], our focus was specifically on investigating the spatial interactions between immune and tumor cells. Therefore, we narrowed our attention to considering only two cell types, i.e., $I = 2$, in the analysis.

References

[1] Wrobel J GT. VectraPolarisData: Vectra Polaris and Vectra 3 multiplex single-cell imaging data R package version 1.0.0.; 2022.

[2] Rendeiro AF, Ravichandran H, Bram Y, Chandar V, Kim J, Meydan C, et al. The spatial landscape of lung pathology during COVID-19 progression. Nature. 2021;593(7860):564–569.

[3] Shannon CE. A mathematical theory of communication. The Bell system technical journal. 1948;27(3):379–423.

[4] Park SY, Gönen M, Kim HJ, Michor F, Polyak K, et al. Cellular and genetic diversity in the progression of in situ human breast carcinomas to an invasive phenotype. The Journal of clinical investigation. 2010;120(2):636–644.

[5] Natrajan R, Sailem H, Mardakheh FK, Arias Garcia M, Tape CJ, Dowsett M, et al. Microenvironmental heterogeneity parallels breast cancer progression: a histology–genomic integration analysis. PLoS medicine. 2016;13(2):e1001961.

[6] Wu HJ, Temko D, Maliga Z, Moreira AL, Sei E, Minussi DC, et al. Spatial intra-tumor heterogeneity is associated with survival of lung adenocarcinoma patients. Cell Genomics. 2022;2(8):100165.

[7] Vu T, Wrobel J, Bitler BG, Schenk EL, Jordan KR, Ghosh D. SPF: a spatial and functional data analytic approach to cell imaging data. PLoS computational biology. 2022;18(6):e1009486.

[8] Yao F, Müller HG, Wang JL. Functional data analysis for sparse longitudinal data. Journal of the American statistical association. 2005;100(470):577–590.

[9] Altieri L, Cocchi D, Roli G. A new approach to spatial entropy measures. Environmental and ecological statistics. 2018;25(1):95–110.

[10] Keren L, Bosse M, Marquez D, Angoshtari R, Jain S, Varma S, et al. A structured tumor-immune microenvironment in triple negative breast cancer revealed by multiplexed ion beam imaging. Cell. 2018;174(6):1373–1387.
